# The suppressor of cytokine signaling 3 regulates glioma stem cell maintenance and immune microenvironment through signal transducer and activator of transcription 3 signaling

**DOI:** 10.1002/ccs3.70041

**Published:** 2026-08-01

**Authors:** Jingtao Wang, Gaolei Hou, Zhaofei Song, Kai Gao, Hongbin Wang, Tao Li

**Affiliations:** ^1^ The Fourth Department of Neurosurgery Affiliated Hospital of Hebei Engineering University Handan China

**Keywords:** apoptosis resistance, glioma stem cells, scRNA‐seq, SOCS3, STAT3 signaling, tumor microenvironment

## Abstract

This study aims to elucidate the role of suppressor of cytokine signaling 3 (SOCS3) in glioma stem cells (GSCs) via single‐cell RNA sequencing (scRNA‐seq), focusing on its regulation of STAT3‐mediated self‐renewal, apoptosis resistance, and tumor microenvironment (TME) remodeling. ScRNA‐seq data from 19 high‐grade glioma patients were analyzed using Seurat, Harmony, and SingleR for clustering, annotation, and SOCS3 stratification (SOCS3‐High: *n* = 4; SOCS3‐Low: *n* = 15). Differential gene analysis, pathway enrichment, and CellChat were employed for TME characterization. In vitro, SOCS3‐overexpressing/silenced GSC11 models were tested via MTT, TUNEL, neurosphere assays, and STAT3 pathway modulation (IL‐6). In vivo, intracranial xenografts in nude mice evaluated tumor growth and survival. SOCS3 was downregulated in GSCs and neurons. SOCS3‐Low GSCs exhibited 777 differentially expressed genes enriched in T‐cell receptor, p53, and JAK‐STAT axis, suppressed T‐cell/microglia infiltration, and promoted oligodendrocyte precursor cell/astrocyte survival. SOCS3 overexpression reduced GSC proliferation, induced apoptosis, inhibited neurosphere formation, and suppressed STAT3 phosphorylation and stemness markers (OCT4/SOX2/NANOG). IL‐6 reactivated STAT3, reversing SOCS3‐mediated tumor suppression. In vivo, SOCS3 overexpression attenuated tumor growth and prolonged survival, counteracted by IL‐6. Low SOCS3 expression contributes to glioma progression by promoting STAT3 activation and an immunosuppressive TME. Targeting the SOCS3‐STAT3 axis may offer therapeutic potential.

## Graphical abstract

We found that SOCS3 deficiency activates STAT3 to enhance glioma stem cell (GSC) stemness and survival. We demonstrated that IL‐6 rescues STAT3 signaling and reverses SOCS3‐mediated tumor suppression. We revealed that SOCS3‐low GSCs exhibit enriched p53 and JAK‐STAT pathway activity. This study provides new mechanistic insights into SOCS3‐STAT3 axis in glioma progression.

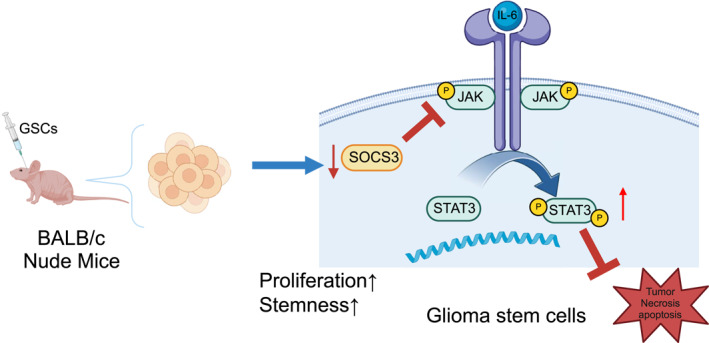

## 1 INTRODUCTION

Gliomas are malignant tumors originating from brain tissue and represent the most common type of malignancies in the adult population, particularly glioblastoma multiforme (GBM).^1^, ^2^ The high mortality, recurrence rate, and drug resistance associated with gliomas make them a significant challenge in neuro‐oncology research.^3^, ^4^ Although surgery, radiotherapy, and chemotherapy are the primary treatments for gliomas, the therapeutic outcomes remain limited due to the tumor's high heterogeneity and the self‐renewal, drug resistance, and migratory properties of glioma stem cells (GSCs).^5^, ^6^, ^7^, ^8^, ^9^ GSCs are considered the “seed” cells of gliomas; they not only contribute to tumor initiation and progression but are also closely associated with tumor recurrence. Studies have shown that GSCs generally exhibit strong drug resistance, and their molecular mechanisms vary across different glioma subtypes.^10^, ^11^, ^12^, ^13^ Therefore, a deeper understanding of the biological characteristics of GSCs and their molecular regulatory mechanisms, especially in the context of the immune microenvironment, cell signaling pathways, and intercellular interactions, is essential for revealing glioma pathogenesis and discovering potential intervention targets.

Suppressor of cytokine signaling 3 (SOCS3) is a key negative regulator of cytokine‐associated signal transduction, exerting essential influences on immune surveillance dynamics and governing processes involved in cellular proliferation and development.^14^, ^15^, ^16^, ^17^ It primarily inhibits the JAK‐STAT axis through a negative feedback mechanism, thereby impacting essential biological processes such as proliferation, differentiation, programmed cell death, and immune function.^18^, ^19^, ^20^ Research has demonstrated that SOCS3 exerts a dual effect in various tumors, both suppressing tumor initiation and promoting tumor progression.^21^, ^22^, ^23^, ^24^ In gliomas, the low expression of SOCS3 is closely linked to malignant tumor progression and poor prognosis in patients.^25^, ^26^ Although SOCS3 has been widely investigated in the context of immune regulation, its specific function and mechanisms in gliomas remain insufficiently defined. Understanding how SOCS3 modulates immune dynamics and intracellular signaling to influence the behavior of GSCs is a pivotal issue in current glioma research.

Signal transducer and activator of transcription 3 (STAT3) is an essential transcriptional regulator within cytokine‐associated pathways, governing multiple biological functions such as proliferation, survival, and cellular self‐renewal.^27^, ^28^, ^29^, ^30^ Research has shown that STAT3 is crucial for the function of GSCs, promoting their self‐renewal, anti‐apoptotic properties, and immune evasion.^31^, ^32^, ^33^ SOCS3 inhibits the activation of the JAK‐STAT axis through a negative feedback mechanism, with STAT3 phosphorylation closely correlated to SOCS3 expression levels.^17^, ^20^ In gliomas, reduced SOCS3 expression may lead to persistent STAT3 activation, thereby enhancing GSCs' survival and proliferative capabilities.^34^, ^35^ Studies have also indicated that the loss or low expression of SOCS3 can drive abnormal activation of the STAT3 signaling pathway, facilitating the malignant transformation and drug resistance of tumor cells.^14^, ^36^, ^37^ The modulation of STAT3 signaling by SOCS3 exerts a substantial influence on GSC function, underscoring the need for deeper mechanistic investigation.

The primary aim of this study is to explore the expression profile of SOCS3 in GSCs and its role in regulating the STAT3 signaling pathway through single‐cell RNA sequencing (scRNA‐seq) analysis. We seek to investigate how SOCS3 modulates STAT3 activity to influence key GSC properties, including self‐renewal, proliferation, apoptosis, and immune evasion, thereby contributing to glioma advancement. We comprehensively investigate the function and regulatory mechanisms of SOCS3 in glioma by integrating scRNA‐seq datasets from the Gene Expression Omnibus with both in vitro and in vivo assays. Our findings may provide a theoretical foundation for targeting SOCS3 as a potential therapeutic strategy and offer new avenues for the precision treatment of glioma. Clinically, SOCS3 could emerge as a novel target for immunotherapy or targeted therapy, particularly for treating GSCs. By modulating the SOCS3/STAT3 signaling pathway, it may enhance therapeutic efficacy, delay tumor recurrence, and improve patient prognosis. Therefore, this study holds significant implications for both fundamental research and the advancement of clinical approaches in glioma therapy.

## 2 MATERIALS AND METHODS

### 2.1 Download of public datasets

scRNA‐seq data derived from glioma tissues were obtained from the public dataset GSE231859, which includes tumor samples from 19 individuals diagnosed with high‐grade gliomas. Preprocessing procedures were implemented utilizing the “Seurat” R package. Cells were filtered based on the criteria: 200 < nFeature_RNA <5000 and mitochondrial gene content (percent.mt) < 20%. The top 2000 most variable genes were selected based on gene expression variance.

As the dataset was sourced from a publicly accessible repository, the study did not necessitate institutional ethical review or acquisition of informed consent.

### 2.2 scRNA‐seq analysis

To manage high‐dimensional transcriptomic data, principal component analysis (PCA) was employed on the 2000 genes exhibiting the most substantial expression fluctuations. Based on the elbow curve generated by Seurat, the top 20 principal components were retained for further downstream analyses. Clustering was performed via the FindClusters function (resolution = 1.0), and Uniform Manifold Approximation and Projection (UMAP) was used to visualize the clusters in two dimensions. Marker genes corresponding to individual clusters were identified through Seurat, and cell‐type annotation was carried out by integrating known lineage‐specific markers with results from the SingleR package and CellMarker database.

Differentially expressed genes (DEGs) for each cluster were identified using the FindAllMarkers function, followed by statistical validation using the Limma framework under the criteria of |log_2_FC| > 0.5 and *p* < 0.05. Resultant gene signatures were visualized via volcano plots constructed using ggplot2. All computational analyses were conducted in R (version 4.3.1).

Cell–cell communication analysis was performed utilizing the “CellChat” package in R.

### 2.3 Enrichment analysis

Gene ontology (GO) enrichment analysis was carried out utilizing the org.Hs.eg.db R package (v3.1.0) as the reference annotation and implemented via the clusterProfiler package (v3.14.3). For Kyoto Encyclopedia of Genes and Genomes (KEGG) pathway analysis, up‐to‐date gene‐pathway associations were retrieved from the KEGG REST API (https://www.kegg.jp/kegg/rest/keggapi.html) and used as the mapping background. The enrichment workflow for KEGG was also conducted with the clusterProfiler package. The minimum gene set size was set to 5 and the maximum was set to 5000, with a significance threshold of *p* < 0.05 and FDR < 0.25. We further performed gene set enrichment analysis (GSEA) on the ranked list of DEGs. Genes were ranked based on their differential expression fold change (logFC). All enrichment analysis results were visualized using the ggplot2 package, including bubble plots (dotplots) for significant pathways and GSEA enrichment curves (enrichment plots) to illustrate significant enrichment terms and their enrichment levels.

### 2.4 Cell culture

The GSC11 (CVCL_DR55), GBM6 (CVCL_DG62), and ReNcell CX (CVCL_E922) cell lines were provided by BioVector. GSCs (GSC11 and GBM6) were cultured in DMEM/F12 medium (21331020, Gibco) enriched with 10% fetal bovine serum (A5670701, Gibco), 1% penicillin/streptomycin (15140122, Gibco), 20 ng/mL epidermal growth factor (EGF) (72528S, CST), and 20 ng/mL bFGF (61977S, CST) to maintain stem cell characteristics. ReNcell CX neural progenitor cells (NPCs) were grown in ReNcell NSC Maintenance Medium (SCM005, Millipore) supplemented with 20 ng/mL EGF and 20 ng/mL bFGF. All cell cultures were maintained in a 37°C incubator with 5% CO_2_.

### 2.5 Cell transfection and grouping

Lentiviral vectors encoding oe‐NC, oe‐SOCS3, sh‐NC, and sh‐SOCS3 were transduced into cells using a packaging system obtained from GeneChem at a viral titer of 1 × 10^8^ TU/mL. Cells were treated with 8 μg/mL polybrene before the virus solution was added. After 48 h post‐transfection, the fresh medium was replaced. Stable cell lines were selected using puromycin (2 μg/mL, Sigma‐Aldrich), and subsequent experiments were carried out 72 h later.^38^ The infection efficiency was confirmed by green fluorescent protein fluorescence and puromycin selection exceeding 90%.

Cell Grouping (*n* = 3): sh‐NC: Cells transfected with lentiviral sh‐NC. sh‐SOCS3: Cells transfected with lentiviral sh‐SOCS3. oe‐NC: Cells transfected with lentiviral oe‐NC. oe‐SOCS3: Cells transfected with lentiviral oe‐SOCS3; oe‐NC + IL‐6 (oe‐NC lentivirus transduced cells treated with 10 ng/mL IL‐6 for 4 h); oe‐SOCS3 + IL‐6 (oe‐SOCS3 lentivirus transduced cells treated with 10 ng/mL IL‐6 for 4 h). oe‐SOCS3 + interleukin 6 (IL‐6): Cells transfected with lentiviral sh‐SOCS3 and treated with 10 ng/mL IL‐6 for 4 h. The SOCS3 knockdown sequences are: sh‐SOCS3‐1: 5′‐CCACCTGGACTCCTATGAGAA‐3′; sh‐SOCS3‐2: 5′‐CCGCTTCGACTGCGTGCTCAA‐3′; sh‐SOCS3‐3: 5′‐CTCCTATGAGAAAGTCACCCA‐3′; The sh‐NC sequence is: 5′‐TTCTCCGAACGTGTCACGT‐3′.

### 2.6 MTT assay

Cell proliferation was assessed using the 3‐(4,5‐Dimethylthiazol‐2‐yl)‐2,5‐diphenyltetrazolium bromide (MTT) assay kit (C0009S, Beyotime). Cells (4 × 10^3^ per well) were seeded in 96‐well plates and cultured for 24, 48, and 72 h. At the designated time points, 20 μL of MTT solution (5 mg/mL) was added to each well, and the plates were incubated at 37°C for 4 h. After incubation, 150 μL of dimethyl sulfoxide (DMSO, D12345, Invitrogen) was added to solubilize the formazan crystals, and the plates were returned to the incubator for an additional 3 h. Absorbance was monitored at 570 nm using a microplate reader (Bio‐Rad). All cell viability experiments were independently repeated three times, each with three technical replicates.

### 2.7 Neurosphere formation assay

To evaluate the self‐renewal capacity of GSC11 cells, 200 cells per well were seeded into a 24‐well ultra‐low attachment plate (Corning) using serum‐free medium. After a 7‐day incubation, neurosphere formation was assessed under a microscope (Leica Microsystems), and both sphere diameter and quantity were analyzed with ImageJ software (NIH).

### 2.8 TUNEL assay

Cell apoptosis was assessed utilizing the One‐Step TUNEL Apoptosis Detection Kit (C1089, Beyotime), which labels fragmented DNA. Cells were fixed with 4% paraformaldehyde (PFA) and then treated with 50 μL of TUNEL detection solution at 37°C for 1 h. DAPI (Invitrogen) was used for nuclear staining. Cell images were captured using a fluorescence microscope (Leica Microsystems), and the apoptosis rate was calculated based on the percentage of cells with positive TUNEL signals. All assays were conducted in triplicate and repeated three times independently.

### 2.9 Immunofluorescence experiment

Cells were fixed in 4% PFA for 10 min and permeabilized with 0.1% Triton X‐100 (P0096, Beyotime), followed by blocking with 1% BSA (ST023, Beyotime) for 1 h. Primary antibodies (details in Table S1) were applied overnight at 4°C, followed by three washes with phosphate‐buffered saline (PBS). Cells were then exposed to species‐appropriate fluorophore‐conjugated secondary antibodies for 1 h at ambient temperature in the dark. Nuclei were counterstained with DAPI, and fluorescence signals were captured using a Leica fluorescence microscope (Leica Microsystems).

For mouse brain sections, a paraffin‐embedded tissue was dewaxed, rehydrated through an ethanol gradient, and subjected to heat‐induced antigen retrieval. After cooling with tap water, sections were washed with 0.5% Triton X‐100 (X100, Sigma) for 10 min and blocked for 1 h in PBS containing 5% BSA (SRE0098, Sigma) and 0.5% Triton X‐100. Primary antibodies were applied overnight at 4°C, followed by PBS washes and incubation with secondary antibodies for 1.5 h at ambient temperature (antibody information is provided in Table S1). After washing, sections were mounted and covered with medium containing DAPI (D9542‐1MG, Sigma). Images were observed under a fluorescence microscope (Leica Microsystems), and quantitative analysis was performed utilizing Fiji software (National Institutes of Health).

### 2.10 Nude mouse model construction

BALB/c nude mice (6–8 weeks old, 17–20 g; strain 401, Beijing Vital River Laboratory Animal Technology Co., Ltd.) were maintained under specific pathogen‐free conditions in individually ventilated cages. Environmental parameters were controlled at 22–25°C with 60%–65% humidity and a 12‐h light/dark cycle. Mice had free access to food and water. After 1 week of environmental adaptation, only physiologically stable animals without observable abnormalities were enrolled in the experiments. All experimental protocols involving animals were reviewed and authorized by the Institutional Animal Care and Use Committee, and carried out in full compliance with relevant institutional policies and national ethical regulations governing laboratory animal welfare.

Mice were anesthetized with sodium pentobarbital (40 mg/kg, P3761, Sigma‐Aldrich) via intraperitoneal injection. After securing the mice in a stereotaxic apparatus (RWD Life Science Co., Ltd.), the surgical area was disinfected, and a hole was drilled in the skull at the pre‐determined injection site. GSCs (glioblastoma stem cells) were infected with lentiviruses carrying either an overexpression of SOCS3 (oe‐SOCS3), control overexpression (oe‐NC), SOCS3 knockdown (sh‐SOCS3), or control knockdown (sh‐NC). Two rounds of transduction were performed at 24‐h intervals. Twenty‐four hours after the second transduction, live cells (2 × 10^5^ GSCs in 5 μL DMEM, 12491015, Gibco) were injected intracranially into the right striatum of the mice to establish an orthotopic glioblastoma model. Sham group mice were injected with 3 μL DMEM at the same coordinates. Thirty minutes after the intracranial injection of GSCs, mice were administered the JAK2/STAT3 pathway activator IL‐6 (HY‐P7063, MCE) via intraperitoneal injection (100 μL). For survival experiments, the mice were monitored for neurological signs until day 180. Tumor growth was assessed by harvesting the brains of GSC‐implanted mice on the same day as the indicated time point.^32^, ^39^, ^40^

The nude mice were randomly divided into eight groups (15 mice per group): Sham (DMEM injection), Model (GSCs injection), oe‐NC (GSCs + oe‐NC), sh‐NC (GSCs + sh‐NC), oe‐SOCS3 (GSCs + oe‐SOCS3), sh‐SOCS3 (GSCs + sh‐SOCS3), IL‐6 (GSCs + IL‐6 antibody), and oe‐SOCS3 + IL‐6 (GSCs + oe‐SOCS3 + IL‐6 antibody).

### 2.11 Hematoxylin and eosin (H&E) staining

Mouse brain tissues were fixed in 4% formaldehyde for 24 h, trimmed, and dehydrated via a graded ethanol and xylene series using an automatic tissue processor. The specimens were then embedded in paraffin, cooled at −20°C, and sectioned at a thickness of 4 μm using a rotary microtome. Paraffin sections were deparaffinized in xylene and rehydrated through descending concentrations of ethanol. Subsequently, sections were stained with hematoxylin (10 min), differentiated in 1% acid ethanol (10 s), rinsed, and counterstained with eosin (5 min). After dehydration and clearing, slides were mounted with neutral resin. Morphological observations were conducted and documented under a bright‐field microscope (Leica Microsystems).

### 2.12 Immunohistochemical staining

Paraffin‐embedded sections were sequentially dewaxed using three environmental‐friendly dewaxing solutions (10 min each), followed by immersion in absolute ethanol (three times, 5 min each), and then rinsed with distilled water. Antigen retrieval was performed by heating sections in citrate buffer (pH 6.0) at boiling temperature for 15 min, followed by cooling and three PBS washes (5 min each, pH 7.4) on a shaker. To inhibit endogenous peroxidase activity, 3% hydrogen peroxide was applied for 25 min in the dark at ambient temperature. After another three PBS washes, sections were blocked with 3% BSA for 30 min at ambient temperature, and then incubated overnight at 4°C with primary antibody against SOCS3 (details listed in Table S1). The next day, sections were washed with PBS and incubated with secondary antibody at ambient temperature for 50 min. 3,3′‐Diaminobenzidine solution (Vector Laboratories) was applied for color development, and the reaction was stopped by rinsing in distilled water. Nuclear counterstaining was performed using hematoxylin for 3 min, followed by differentiation (5 s) and bluing. Sections were then dehydrated through graded ethanol (75%, 85%, absolute ethanol ×2, 5 min each), n‐butanol (5 min), and xylene (5 min), air‐dried and coverslipped. Images were captured under a light microscope.

### 2.13 Reverse transcription quantitative polymerase chain reaction (RT‐qPCR) experiment

Total RNA was isolated from cultured cells and tissue specimens using commercial RNA purification kits (catalog numbers 12183020 and 12183018A, Thermo Fisher Scientific). For reverse transcription, 1 μg of RNA template was converted into complementary DNA (cDNA) utilizing the First Strand cDNA Synthesis Kit (K1622, Fermentas). Quantitative PCR was performed using the BeyoFast™ SYBR Green One‐Step qRT‐PCR Kit (D7268S, Beyotime) on the ABI StepOnePlus or 7500 Real‐Time PCR systems (Applied Biosystems, Thermo Fisher). Gene expression levels were calculated by applying the 2−ΔΔCt method, with β‐actin employed as the endogenous reference gene for normalization. All reactions were carried out in triplicate to ensure reproducibility. The RT‐qPCR reactions were conducted on the StepOnePlus system (Applied Biosystems). Details of the primer sequences utilized in the experiment are listed in Table S2. All reagents and materials used in the experiment were purchased from ServiceBio.

### 2.14 Western blot (WB)

Proteins from tumor tissues and cells were extracted using the Tissue Protein Extraction Kit (EX2171, Solarbio) and Cell Protein Extraction Kit (EX2170, Solarbio), respectively. Protein concentrations were determined by BCA assay (BCA1‐1KT, Sigma). Equal amounts of protein (20 μg per lane) were separated on 10%–12% SDS‐PAGE gels and transferred to PVDF membranes (Millipore). Membranes were blocked with 5% BSA for 2 h at ambient temperature, and then incubated overnight at 4°C with primary antibodies (see Table S1), followed by HRP‐conjugated secondary antibodies (goat anti‐rabbit IgG H&L, ab97051; goat anti‐mouse IgG, ab205719; Abcam, 1:2000). Signals were visualized using ECL reagent (Thermo Fisher), and band intensities were analyzed using ImageJ.

### 2.15 Statistical analysis

All statistical analyses were carried out utilizing GraphPad Prism 9.0 (GraphPad Software). Quantitative data were presented as mean ± standard deviation. Comparisons between two groups were conducted using unpaired two‐tailed Student's *t*‐tests, whereas multiple group comparisons were assessed by one‐way analysis of variance. Categorical data were presented as rates or percentages and analyzed using the chi‐square test. A *p*‐value of <0.05 was considered statistically significant.

## 3 RESULTS

### 3.1 SOCS3 regulation of cellular heterogeneity and signaling pathway activity in glioma

Our previous study established that SOCS3 exerts a critical regulatory function in gliomas by suppressing the JAK‐STAT axis.^41^ To explore its single‐cell regulatory effects, we analyzed a scRNA‐seq dataset derived from 19 high‐grade glioma patients (GSE231859). After standard preprocessing and batch correction, a total of 14,078 high‐quality cells expressing 19,176 genes were retained (Figure S1A). Following normalization and identification of highly variable genes, PCA was applied for initial dimensionality reduction (Figure S1B). PCA visualization revealed inter‐sample batch effects (Figure S1C), which were effectively corrected using the Harmony algorithm (Figure S1D,E). Subsequent UMAP analysis grouped the cells into 25 distinct clusters (Figure S1F).

**FIGURE 1:**
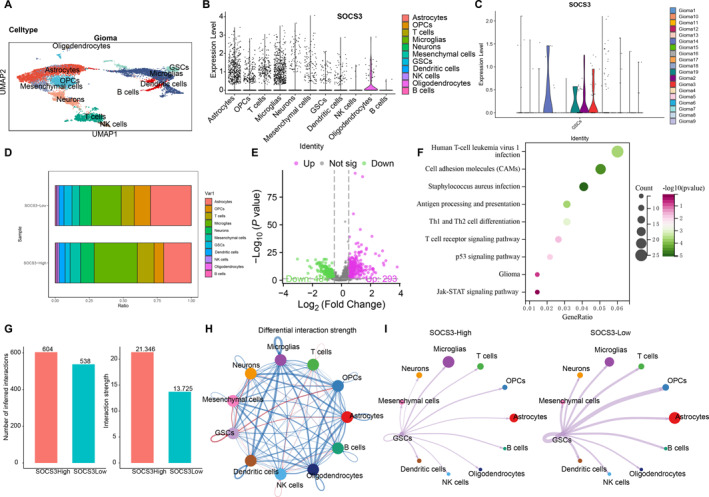
Investigation of the effect of SOCS3 on cellular heterogeneity and signaling pathway activity in glioma. (A) UMAP clustering‐based visualization of cell annotation groupings; (B) violin plot showing SOCS3 expression across 11 cell types; (C) SOCS3 expression in 19 samples from selected neural stem cells; (D) bar chart of the cell type proportions in two sample groups; (E) volcano plot showing differential expression analysis of SOCS3 in neural stem cells, where purple indicates significantly upregulated genes, green indicates significantly downregulated genes, and gray indicates genes with no significant difference; (F) KEGG enrichment analysis of DEGs; (G) comparison of total signaling pathway interactions and interaction strength between SOCS3‐High and SOCS3‐Low groups; (H) comparison of interaction strength between cells in SOCS3‐High and SOCS3‐Low groups, with blue indicating downregulation and red indicating upregulation; (I) interaction network specifically between neural stem cells and other cell types. SOCS3‐High: *n* = 4; SOCS3‐Low: *n* = 15. DEGs, differentially expressed genes; KEGG, Kyoto encyclopedia of genes and genomes; SOCS3, suppressor of cytokine signaling 3; UMAP, uniform manifold approximation and projection.

We then employed the Bioconductor/R package “SingleR” combined with manual annotation to automatically annotate the 25 cell clusters, leading to the identification of 11 distinct cell types (Figure 1A). To validate the accuracy of the clustering and annotation, a heatmap was generated displaying the top five marker genes with the highest expression levels for each cell type (Figure S2A). Among these, GSCs were successfully identified based on canonical neural stem cell signatures, consistent with their proposed origin from normal NPCs.^42^ Further analysis of SOCS3 revealed its lower expression levels in GSCs and neurons (Figure 1B). Additionally, higher expression levels were observed in four samples (Glioma2, Glioma3, Glioma14, and Glioma19), leading to the classification of the 19 samples into two groups: SOCS3‐High and SOCS3‐Low (Figure 1C).

We reanalyzed the cell distribution in both groups (Figure 1D, Figure S2B, Table S3). We observed a significant reduction in the number of T cells and microglia in the SOCS3‐Low group compared to the SOCS3‐High group, whereas the numbers of oligodendrocyte precursor cells (OPCs) and astrocytes increased. These findings suggest that low expression of SOCS3 inhibits the infiltration of T cells and microglia, potentially leading to an immunosuppressive environment conducive to tumor escape. Additionally, SOCS3 downregulation promotes the survival and expansion of OPCs and astrocytes, which may contribute to the tumor's cellular origins.

Differential expression analysis of GSCs revealed 777 DEGs, including 293 upregulated and 484 downregulated genes (Figure 1E, Table S4). GO enrichment analysis revealed that, within the BP category, the DEGs were primarily involved in nuclear‐transcribed mRNA catabolic process, protein localization to the endoplasmic reticulum (ER), and ER targeting (Figure S3A). In the cellular component analysis, vesicle, extracellular vesicle, and extracellular organelle were prominently associated (Figure S3B). Molecular function analysis showed significant enrichment in enzyme binding, protein complex binding, and enzyme regulator activity (Figure S3C). KEGG pathway enrichment analysis revealed that these DEGs were predominantly involved in immune and oncogenic signaling cascades, including HTLV‐1 infection, Th1/Th2 cell differentiation, T cell receptor‐mediated signaling, p53 pathway activity, and JAK‐STAT signal transduction (Figure 1F). Moreover, GSEA further supported the involvement of SOCS3 in regulating the T cell receptor, p53, and JAK‐STAT signaling pathways in glioma (Figure S3D–F).

In the study of SOCS3's regulatory role in cell–cell signaling, we utilized the “CellChat” software package to reveal communication between different cell phenotypes. Comparative analysis between SOCS3‐High and SOCS3‐Low groups revealed that the SOCS3‐High group exhibited more extensive and stronger intercellular signaling interactions (Figure 1G). Further analysis of interaction strength revealed that, with the exception of increased interactions between GSCs and OPCs in the SOCS3‐Low group, all other cell–cell interactions were attenuated (Figure 1H). An in‐depth analysis of the SOCS3‐Low group revealed distinct interaction patterns between cells, with a particular focus on the impact of GSCs on other cell types. We found that in the SOCS3‐Low group, GSCs generally had a stronger influence on other cells particularly on OPCs (Figure 1I).

Comprehensive analysis of scRNA‐seq data revealed that SOCS3 expression was closely associated with distinct cellular subpopulations and significantly impacted intercellular communication and transcriptional profiles within the tumor microenvironment (TME). Further analysis of DEGs and functional enrichment revealed that SOCS3 may influence glioma progression through canonical immune and tumor‐associated pathways, including T cell receptor, p53, and JAK‐STAT axis.

### 3.2 Overexpression of SOCS3 inhibits the proliferation and self‐renewal of GSCs

**FIGURE 2:**
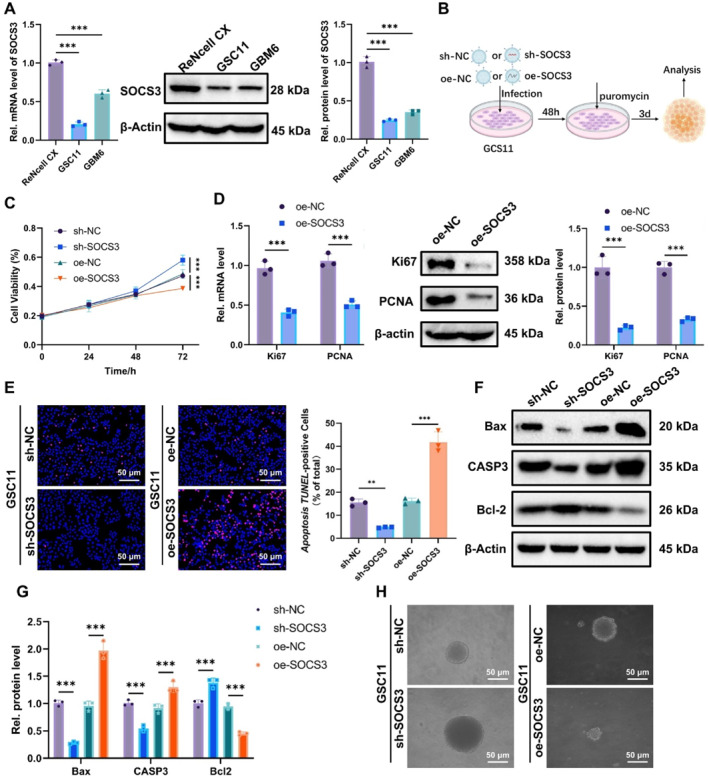
Investigation of the effect of SOCS3 on the self‐renewal and anti‐apoptotic abilities of GSCs. (A) mRNA and protein expression levels of SOCS3 in GSCs (GSC11, GBM6) and NPCs (ReNcell CX) cells, detected by RT‐qPCR and WB; (B) schematic diagram illustrating the process for establishing stable GSC11 cell lines; (C) MTT assay showing the proliferation of GSC11 cells after SOCS3 overexpression or knockdown; (D) mRNA and protein expression levels of proliferation markers Ki67 and PCNA following SOCS3 overexpression, detected by RT‐qPCR and WB; (E) TUNEL staining detecting apoptosis levels in GSC11 cells after SOCS3 overexpression or knockdown, scale bar = 50 μm; (F, G) WB analysis of protein expression levels of Bax, Bcl‐2, and Caspase‐3 in GSC11 cells after SOCS3 overexpression or knockdown; (H) neurosphere formation assay assessing the effect of SOCS3 overexpression or knockdown on the self‐renewal capacity of GSC11, scale bar = 50 μm. **A comparison between the two groups, *p* < 0.01; ***p < 0.001. Cell experiments were repeated three times. GSCs, glioma stem cells; MTT, 3‐(4,5‐dimethylthiazol‐2‐yl)‐2,5‐diphenyltetrazolium bromide; NPCs, neural progenitor cells; PCNA, proliferating cell nuclear antigen; RT‐qPCR, reverse transcription quantitative polymerase chain reaction; SOCS3, suppressor of cytokine signaling 3; WB, western blot.

We subsequently investigated the effect of SOCS3 on GSCs in vivo. Initially, we assessed the expression levels of SOCS3 in GSCs (GSC11, GBM6) and normal NPCs (ReNcell CX) using RT‐qPCR and WB analysis. The results showed that SOCS3 expression was significantly lower in GSCs compared to NPCs, with the lowest expression observed in GSC11 cells (Figure 2A). Therefore, we chose GSC11 for subsequent experiments. Next, we constructed lentiviral vectors for SOCS3 overexpression (oe‐SOCS3) and knockdown (sh‐SOCS3), and successfully transduced GSC11 cells. After puromycin selection, stable cell lines were established (Figure 2B). The modulation of SOCS3 expression was confirmed by RT‐qPCR and WB (Figure S4), with sh‐SOCS3‐1 showing the highest knockdown efficiency. Thus, sh‐SOCS3‐1 was selected for further experiments.

MTT assays revealed that GSC11 cells with oe‐SOCS3 exhibited a markedly lower proliferation rate than those in the oe‐NC control group. In contrast, sh‐SOCS3 significantly enhanced proliferative capacity relative to sh‐NC cells (Figure 2C). RT‐qPCR and WB analyses of the proliferation markers Ki67 and proliferating cell nuclear antigen (PCNA) (Figure 2D) further confirmed that SOCS3 overexpression significantly inhibited GSC11 proliferation. We then evaluated the apoptosis levels in each group. TUNEL staining demonstrated a substantial increase in apoptotic cells in the oe‐SOCS3 group compared to controls, whereas silencing SOCS3 resulted in reduced apoptosis (Figure 2E). WB analysis further confirmed that, compared to the oe‐NC group, SOCS3 overexpression upregulated the expression of pro‐apoptotic proteins Bax and Caspase‐3 while downregulating the anti‐apoptotic protein Bcl‐2 expression. Conversely, SOCS3 knockdown in the sh‐SOCS3 group downregulated Bax and Caspase‐3 and upregulated Bcl‐2 expression (Figure 2F,G). Additionally, neurosphere formation assays demonstrated that SOCS3 overexpression significantly inhibited the neurosphere formation efficiency of GSC11 cells, whereas SOCS3 knockdown enhanced the self‐renewal capacity of GSC11 cells (Figure 2H).

These findings suggest that SOCS3 functions as a negative regulator of GSC self‐renewal and proliferation while facilitating apoptosis in GSCs.

### 3.3 SOCS3 inhibits STAT3 phosphorylation and downregulates key transcription factors in GSCs

**FIGURE 3:**
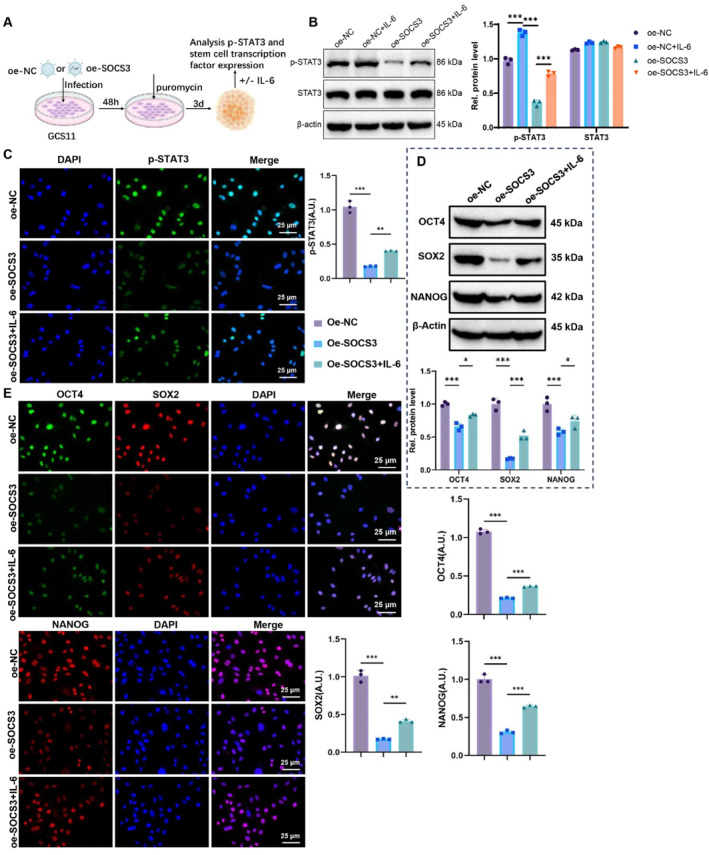
Effects of SOCS3 on STAT3 phosphorylation and stem cell transcription factor expression. (A) Schematic of the experimental workflow for constructing SOCS3‐overexpressing stable cell lines with or without IL‐6 treatment; (B) WB analysis of the effect of SOCS3 overexpression and IL‐6 treatment on STAT3 phosphorylation in GSC11 cells; (C) immunofluorescence staining to assess the impact of SOCS3 overexpression and IL‐6 treatment on phospho‐STAT3 in GSC11 cells, scale bar = 25 μm; (D) WB analysis of the effect of SOCS3 overexpression and IL‐6 treatment on the expression of OCT4, SOX2, and NANOG in GSC11 cells; (E) immunofluorescence staining to evaluate the impact of SOCS3 overexpression and IL‐6 treatment on the stem cell transcription factors OCT4, SOX2, and NANOG in GSC11 cells, scale bar = 25 μm. *p < 0.05, **p < 0.01, ***p < 0.001, based on comparisons between two groups. Cell experiments were repeated three times. SOCS3, suppressor of cytokine signaling 3; WB, western blot.

To investigate the specific mechanism by which SOCS3 inhibits the self‐renewal capacity of GSCs, we transfected GSC11 cells with an oe‐SOCS3 lentivirus and treated the cells with an agonist of the JAK2/STAT3 pathway (IL‐6) (Figure 3A). WB analysis demonstrated a significant reduction in phosphorylated STAT3 (p‐STAT3) levels in the oe‐SOCS3 group relative to the oe‐NC control (Figure 3B). In contrast, p‐STAT3 expression was markedly elevated in the oe‐SOCS3 + IL‐6 group compared to the oe‐SOCS3 group, and similarly increased in the oe‐NC + IL‐6 group compared to oe‐NC. Immunofluorescence staining results were consistent with these findings (Figure 3C). Further investigation of pluripotency‐associated transcription factors revealed that SOCS3 overexpression significantly downregulated OCT4, SOX2, and NANOG expression at both the protein and immunofluorescence levels. After IL‐6 treatment, the expression of these transcription factors was upregulated in the oe‐SOCS3 + IL‐6 group (Figure 3D,E).

These results suggest that SOCS3 inhibits the phosphorylation of STAT3, thereby reducing the expression of stem cell transcription factors and suppressing the self‐renewal capacity of GSC11 cells.

### 3.4 Reactivation of STAT3 reverses the inhibition of GSC proliferation and anti‐apoptotic capacity induced by SOCS3 overexpression

**FIGURE 4:**
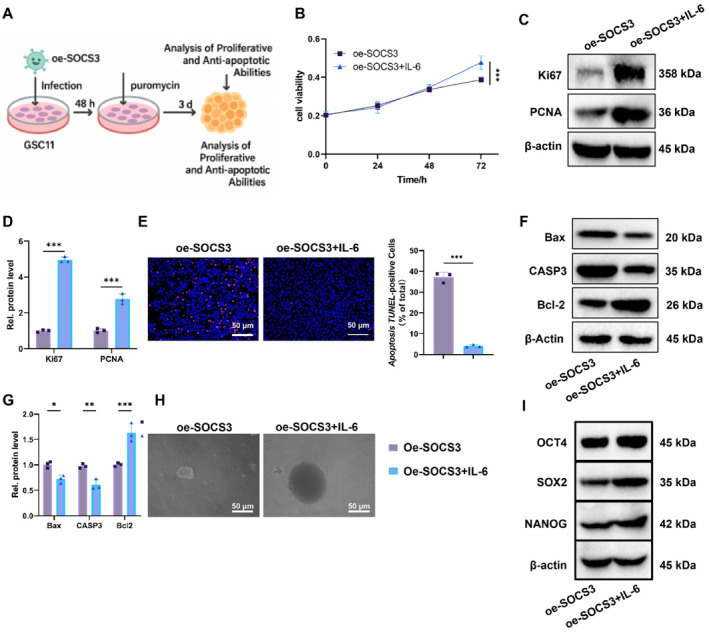
STAT3 activation reverses the effects of SOCS3 overexpression on proliferation and anti‐apoptotic ability of GSCs. (A) Schematic diagram of the experimental process for assessing the effects of SOCS3 overexpression and IL‐6 treatment on GSC proliferation and anti‐apoptotic ability; (B) MTT assay to evaluate the effect of SOCS3 overexpression and IL‐6 treatment on GSC11 proliferation; (C, D) WB analysis of the impact of SOCS3 overexpression and IL‐6 treatment on proliferation markers Ki67, PCNA, and apoptosis‐related proteins Bax, Bcl‐2, and Caspase‐3; (E) TUNEL staining to assess the apoptotic level of GSC11 after SOCS3 overexpression and IL‐6 treatment (scale bar = 50 μm); (F, G) WB analysis of the effects of SOCS3 overexpression and IL‐6 treatment on Bax, Caspase‐3, and Bcl‐2 levels; (H) neurosphere formation assay to evaluate the effect of SOCS3 overexpression and IL‐6 treatment on GSC11 self‐renewal capacity (scale bar = 50 μm); (I) western blot analysis of the expression levels of stemness markers OCT4, SOX2, and NANOG after SOCS3 overexpression and IL‐6 treatment. *Comparison between two groups, *p* < 0.05, **p < 0.01, ***p < 0.001. All cell experiments were performed in triplicate. GSCs, glioma stem cells; MTT, 3‐(4,5‐dimethylthiazol‐2‐yl)‐2,5‐diphenyltetrazolium bromide; PCNA, proliferating cell nuclear antigen; SOCS3, suppressor of cytokine signaling 3; WB, western blot.

We further investigated the impact of SOCS3 overexpression and IL‐6 treatment on the proliferation and anti‐apoptotic capacity of GSCs (Figure 4A). MTT assay results revealed that IL‐6 stimulation significantly restored proliferative capacity in the oe‐SOCS3 + IL‐6 group compared to the oe‐SOCS3 group (Figure 4B). WB analysis of the proliferation markers Ki67 and PCNA supported these findings, demonstrating that IL‐6 activation effectively reversed the anti‐proliferative effects induced by SOCS3 overexpression (Figure 4C,D). These results indicate that JAK2/STAT3 pathway reactivation rescues the reduced proliferation of GSC11 cells caused by SOCS3.

Additionally, TUNEL staining was used to assess apoptosis levels in various groups, and the results revealed a significant reduction in apoptosis after IL‐6 treatment relative to the oe‐SOCS3 group (Figure 4E). Consistently, WB analysis demonstrated that IL‐6 stimulation decreased the expression of pro‐apoptotic markers Bax and Caspase‐3 while upregulating the anti‐apoptotic protein Bcl‐2 (Figure 4F,G). In the neurosphere formation assay, IL‐6 activation restored the self‐renewal capacity of GSC11 cells compared to the SOCS3 overexpression group (Figure 4H). Compared to the oe‐SOCS3 group, IL‐6 stimulation led to a marked upregulation of stemness‐associated transcription factors OCT4, SOX2 and NANOG, indicating that IL‐6 enhances stemness (Figure 4I).

These results demonstrate that STAT3 activation effectively reverses the negative effects of SOCS3 overexpression by restoring stem cell self‐renewal and anti‐apoptotic abilities.

### 3.5 Targeting SOCS3 significantly inhibits the tumor growth of GSCs and prolongs survival in nude mice

**FIGURE 5:**
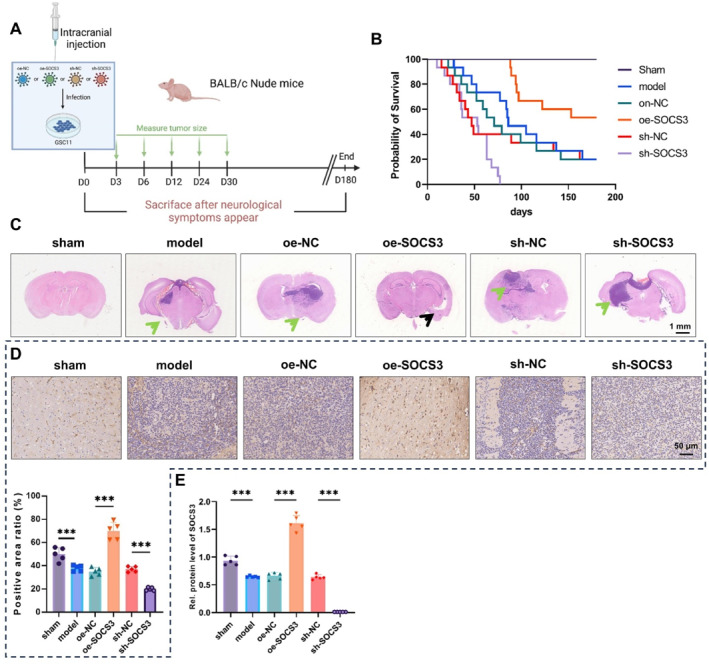
Targeting SOCS3 significantly inhibits tumor growth of GSCs and extends survival in nude mice. (A) Experimental workflow of tumorigenicity and survival study in a mouse intracranial GBM model targeting SOCS3; (B) survival analysis of GSCs intracranial allograft tumor mouse models in each group (*n* = 15); (C) H&E staining of brain sections from BALB/c nude mice collected after GSCs implantation. Green arrows indicate large tumor tissue invading normal tissue. Black arrows indicate small tumor nodules, scale bar = 1 mm; (D) SOCS3 immunohistochemical staining of brain specimens from intracranial orthotopic tumor‐bearing mice in each group, scale bar = 50 μm; (E) WB analysis of SOCS3 expression levels in brain tissues of mice from each group. ***p < 0.001, *n* = 5. GBM, glioblastoma multiforme; GSCs, glioma stem cells; H&E, hematoxylin and eosin; SOCS3, suppressor of cytokine signaling 3; WB, western blot.

Given the pivotal role of SOCS3 in regulating GSC stemness, we hypothesized that SOCS3 may influence glioblastoma progression in vivo. To test this, we established an orthotopic xenograft model using GSC11 cells in nude mice to evaluate the impact of SOCS3 modulation on tumorigenicity and survival (Figure 5A). Kaplan–Meier survival analysis demonstrated that mice in the Model group had significantly reduced survival compared to the Sham group, whereas SOCS3 overexpression markedly prolonged survival. In contrast, SOCS3 knockdown further reduced survival (Figure 5B). Histopathological examination with H&E staining revealed densely packed, actively proliferating tumor cells in the Model group with no necrosis, whereas tumors from the oe‐SOCS3 group exhibited prominent central necrosis, apoptotic features, cellular deformation, and increased mitotic figures. In contrast, SOCS3 knockdown promoted aggressive tumor growth without evident necrotic zones or immune cell infiltration (Figure 5C). Immunohistochemistry and WB analyses showed reduced SOCS3 expression in the Model group relative to Sham. In contrast, SOCS3 levels were upregulated in the oe‐SOCS3 group relative to the oe‐NC group, whereas SOCS3 expression was markedly reduced in the sh‐SOCS3 group relative to the sh‐NC group (Figure 5D,E). Moreover, expression levels of core stemness markers OCT4, SOX2, and NANOG were downregulated in the oe‐SOCS3 group relative to controls, as shown by immunohistochemistry (Figure S5).

In summary, the findings indicate that SOCS3 suppresses the growth of GSCs and reduces their tumorigenicity in vivo, thereby extending the survival of GBM xenografted nude mice.

### 3.6 Activation of STAT3 significantly reverses the effect of SOCS3 on GSC tumor growth and survival of nude mice

**FIGURE 6:**
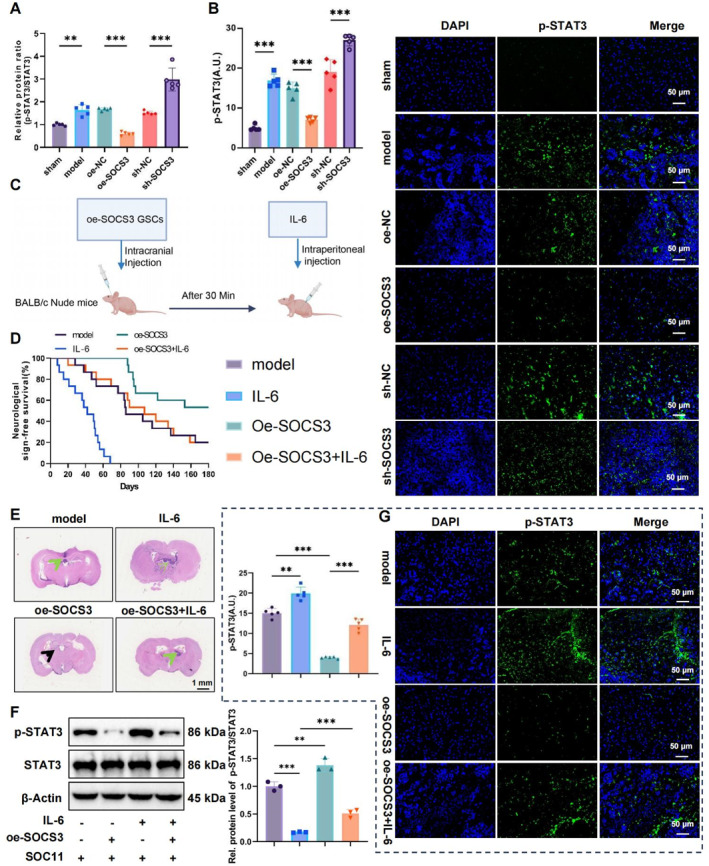
SOCS3 regulates GSC tumor growth and nude mouse survival through the JAK‐STAT signaling pathway. (A) WB analysis of the phosphorylation levels of STAT3 in tumor tissues from different experimental groups; (B) immunofluorescence staining showing changes in the expression of phosphorylated STAT3 protein in tumor tissues from different experimental groups. Scale bar = 50 μm; (C) schematic diagram illustrating the experimental protocol, showing the treatment of oe‐SOCS3 mouse models with the JAK2/STAT3 pathway activator IL‐6; (D) survival analysis of intracranial xenograft models in mGSCs‐transplanted mice (*n* = 15); (E) H&E staining of brain sections from BALB/c nude mice collected after GSCs implantation. Green arrows indicate large tumor masses invading normal tissue. Black arrows indicate small tumor nodules. Scale bar = 20 μm; (F) WB analysis of the phosphorylation levels of STAT3 in tumor tissues from different groups of mice; (G) immunofluorescence staining showing the expression and localization of phosphorylated STAT3 protein in tumor tissues from different groups. Scale bar = 50 μm; **p < 0.01, ***p < 0.001, *n* = 5. GSC, glioma stem cell; H&E, hematoxylin and eosin; SOCS3, suppressor of cytokine signaling 3; WB, western blot.

Subsequent WB analysis (Figure 6A) demonstrated that STAT3 phosphorylation was markedly increased in tumor tissues from the Model group relative to the Sham group. However, SOCS3 overexpression substantially suppressed STAT3 phosphorylation relative to the oe‐NC group. Additionally, tumor tissues from the sh‐SOCS3 group exhibited elevated STAT3 phosphorylation compared to the sh‐NC group, a finding further validated by immunofluorescence staining consistent with the WB results (Figure 6B).

To further investigate whether SOCS3 regulates tumor growth and survival via the STAT3 signaling pathway, we treated oe‐SOCS3 GSC intracranial xenograft mice with an activator of the JAK2/STAT3 pathway (IL‐6) (Figure 6C). Survival analysis revealed that IL‐6 treatment reduced the survival time compared to the Model group (Figure 6D). Moreover, survival was markedly shortened in the oe‐SOCS3 + IL‐6 group relative to the oe‐SOCS3 group.

Histological analysis by H&E staining demonstrated that tumors in the oe‐SOCS3 + IL‐6 group exhibited indistinct boundaries, increased structural disorganization, and decreased apoptotic features compared to those in the oe‐SOCS3 group (Figure 6E). WB and immunofluorescence analyses further revealed that STAT3 phosphorylation was elevated in the IL‐6 group relative to the Model group, and in the oe‐SOCS3 + IL‐6 group compared to the oe‐SOCS3 group (Figure 6F,G).

These findings indicate that the activation of STAT3 significantly reverses the effect of SOCS3 on GSC tumor growth and the survival of nude mice.

## 4 DISCUSSION

This study reveals a close association between the low expression of SOCS3 in GSCs and the malignant progression of glioma. As a key negative regulator, SOCS3 plays a crucial role in modulating immune responses and cell proliferation in various tumors. Previous studies have shown that SOCS3 regulates immune response and tumor progression through inhibition of the JAK/STAT pathway.^43^, ^44^, ^45^ However, research on the role of SOCS3 in gliomas, especially its function in GSCs, is limited. Most studies have focused on the immune‐regulatory role of SOCS3, suggesting that it suppresses tumor progression by affecting immune cell infiltration and immune evasion mechanisms. In contrast, our single‐cell transcriptomic analysis elucidates the specific role of SOCS3 in GSCs, filling a research gap and offering a new perspective on its function in glioma.

In this study, we identified SOCS3 as a pivotal regulator of GSC proliferation and self‐renewal by regulating the JAK/STAT3 signaling pathway. STAT3, a key transcription factor in cytokine signaling, has been widely implicated in tumorigenesis and cancer progression.^46^, ^47^, ^48^ Persistent activation of STAT3 is considered a core mechanism that promotes GSC self‐renewal, apoptosis resistance, and immune evasion in various tumors, including gliomas.^32^, ^49^, ^50^, ^51^ SOCS3 inhibits STAT3 phosphorylation through a negative feedback mechanism, thereby suppressing its transcriptional activity and impacting tumor cell proliferation and survival.^36^, ^52^ Our findings are consistent with previous studies, which have shown that the loss or downregulation of SOCS3 leads to the overactivation of STAT3 and promotes malignant transformation. In contrast to prior studies that primarily emphasized the immune‐regulatory functions of SOCS3, our findings highlight its direct role in governing GSC proliferation and self‐renewal, thereby expanding the functional scope of SOCS3.

This study also found that the low expression of SOCS3 significantly inhibited the infiltration of T cells and microglia, thereby creating an immunosuppressive microenvironment that promotes glioma initiation and progression. These findings align with previous studies indicating that SOCS3 serves as a critical regulator of the immune landscape within the TME.^53^ In the context of tumor immune evasion, low expression of SOCS3 typically leads to the upregulation of immunosuppressive cytokines, which inhibit the effective infiltration of immune cells, thus enhancing tumor survival and expansion. Numerous studies have demonstrated that SOCS3's role in immune response extends beyond its regulation of immune cell infiltration; it is also closely involved in tumor‐associated inflammatory responses.^23^ By focusing on GSCs, this study demonstrates that reduced SOCS3 expression contributes to the formation of an immunosuppressive TME, thereby promoting glioma progression and cell survival. These findings provide novel insights into the immunomodulatory functions of SOCS3 and highlight its potential as a promising therapeutic target for glioma immunotherapy.

Our experimental data demonstrate that SOCS3 overexpression markedly suppresses GSC proliferation and tumor growth by downregulating the phosphorylation of STAT3. This finding is consistent with results from various other tumor types, suggesting that SOCS3, as a negative regulator, can attenuate tumor progression by inhibiting excessive activation of cytokine signaling pathways. However, unlike previous studies that primarily focus on the immune regulatory role of SOCS3 overexpression, this study further explores its impact on GSC self‐renewal and proliferation, revealing that SOCS3 directly affects the biological characteristics of GSCs. We propose that this discovery offers a new perspective on SOCS3 as a therapeutic target for glioma, particularly in therapies targeting GSCs, where SOCS3 modulation may represent a novel treatment strategy.

This study demonstrates that SOCS3 significantly influences GSC self‐renewal and anti‐apoptotic capacity by modulating the STAT3 signaling pathway, suggesting its potential clinical value in glioma therapy. Immune evasion and drug resistance remain major challenges in clinical glioma treatment. SOCS3's negative regulation of the STAT3 pathway may offer a new target to address these issues. Previous research has shown that targeting the STAT3 pathway effectively inhibits tumor progression.^46^, ^54^ However, direct investigations targeting SOCS3 remain limited. We propose that SOCS3‐targeted therapies may not only suppress GSC proliferation but also enhance immunotherapeutic efficacy by restoring antitumor immune responses. In the future, SOCS3 may offer new treatment strategies for glioma, particularly in the context of precision medicine and immunotherapy.

Although this study highlights the critical role of SOCS3 in GSCs, several limitations remain. First, although the scRNA‐seq data provide a high‐resolution analysis of cellular heterogeneity, validation with larger patient cohorts is still necessary. Second, this study primarily utilized the GSC11 cell line and nude mouse models and did not include a broader range of GSC subtypes. Future studies should expand to GSC models derived from different glioma subtypes and clinical patients. Third, the impact of SOCS3 on the glioma immune microenvironment has not been fully verified by in vivo experiments. Future research can combine tumor immune microenvironment remodeling strategies to further assess the potential application of SOCS3 in immunotherapy. Furthermore, single‐cell analysis revealed significant interactions between OPCs and SOCS3‐low GSCs. Subsequent studies may explore the additional mechanisms of SOCS3 in glioma with a focus on OPCs. Finally, as this study is primarily based on in vitro and in vivo models, further investigations are warranted to evaluate its clinical translational potential. Future work should validate the association between SOCS3 and patient prognosis in clinical samples and assess the feasibility of targeting SOCS3 for therapy.

## 5 CONCLUSION

Based on the results above, we can preliminarily conclude the following: Low expression of SOCS3 promotes an immunosuppressive microenvironment, enhancing the self‐renewal and anti‐apoptotic abilities of GSCs, thus driving glioma progression. This study is the first to systematically analyze the role of SOCS3 in glioma at the single‐cell level, revealing its regulation of GSC stemness and anti‐apoptotic properties through the JAK‐STAT3 axis. This research expands our understanding of the glioma microenvironment and cellular heterogeneity and provides significant evidence for the potential of SOCS3 as a therapeutic target. Inhibition of the STAT3 pathway or enhancement of SOCS3 expression may represent effective strategies to interfere with glioma growth and overcome treatment resistance, offering new insights into precision therapy for glioma.

## AUTHOR CONTRIBUTIONS

Jingtao Wang and Gaolei Hou contributed equally to data analysis, in vitro and in vivo experiments, and manuscript drafting. Zhaofei Song and Kai Gao assisted with scRNA‐seq analysis and bioinformatics interpretation. Hongbin Wang supported animal studies and data curation. Tao Li conceived and supervised the study, secured funding, and critically revised the manuscript. All authors read and approved the final version of the manuscript.

## CONFLICT OF INTEREST STATEMENT

The authors declare no conflicts of interest.

## ETHICS STATEMENT

All animal experiments were approved by the Animal Ethics Committee of Affiliated Hospital of Hebei Engineering University.

## PATIENT CONSENT STATEMENT

Not applicable.

## PERMISSION TO REPRODUCE MATERIAL FROM OTHER SOURCES

None.

## CLINICAL TRIAL REGISTRATION

Not applicable.

## Supporting information

Supporting Information S1

## Data Availability

All data can be provided as needed.
