# Supplementary material for: The suppressor of cytokine signaling 3 regulates glioma stem cell maintenance and immune microenvironment through signal transducer and activator of transcription 3 signaling
Source: J Cell Commun Signal. 2026 Aug 1;20(3):e70041. doi: 10.1002/ccs3.70041 (PMC13428608; doi:10.1002/ccs3.70041)
Supplement: Supplementary file 1 — Supporting Information S1 [file CCS3-20-e70041-s001.docx]

**
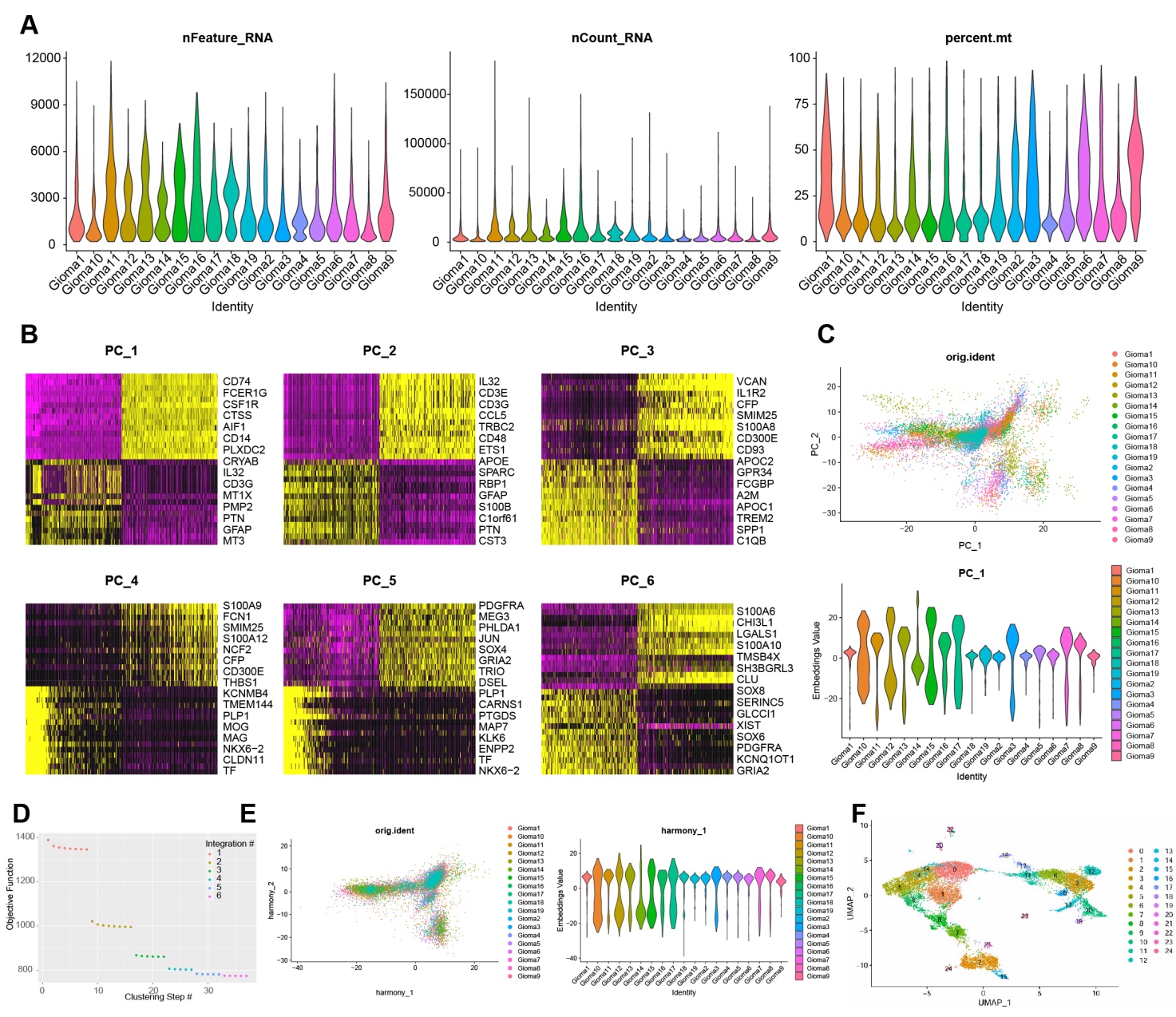
**

**Figure S1. Quality Control, Filtering, and PCA of scRNA-seq Data.**

Note: (A) Violin plots showing the number of genes per cell (nFeature_RNA), the number of mRNA molecules (nCount_RNA), and the percentage of mitochondrial genes (percent.mt) in the scRNA-seq data; (B) Heatmap of the top 20 most strongly correlated genes in PC_1–PC_6 from the PCA, with yellow indicating upregulation and purple indicating downregulation; (C) Left panel shows the distribution of cells in PC_1 and PC_2 before batch correction, with each point representing a single cell. The right panel shows violin plots of the distribution in PC_1 and PC_2; (D) Harmony batch correction process plot, with the x-axis representing the number of interaction impacts; (E) Left panel shows the distribution of cells in PC_1 and PC_2 after Harmony batch correction, with each point representing a single cell. The right panel shows the violin plot after correction. Glioma: n=19; (F) UMAP clustering results with cells partitioned into 25 distinct clusters.

**
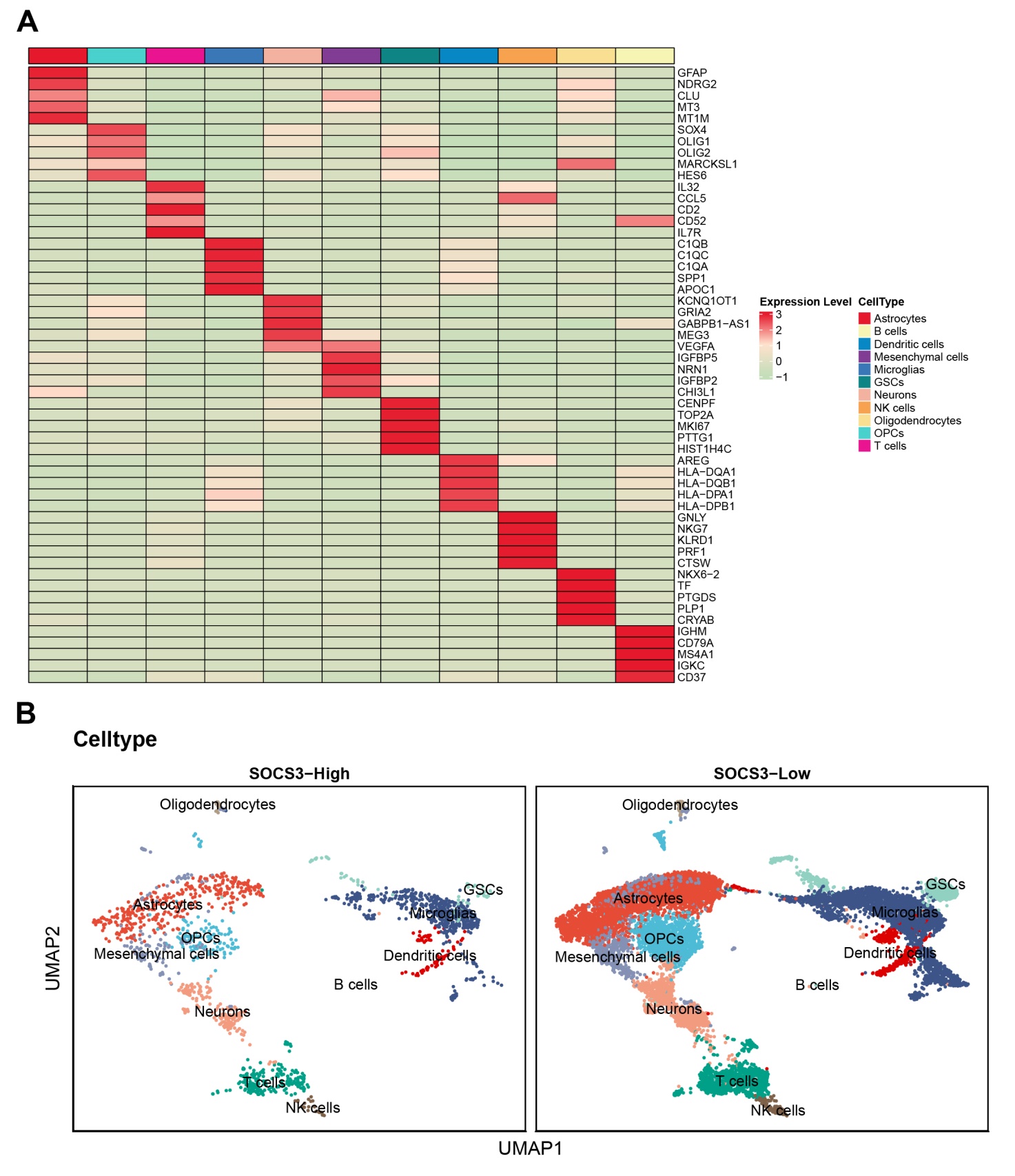
**

**Figure S2. scRNA-seq Data Cell Clustering.**

Note: (A) Heatmap showing the correlation of the top 5 expressed genes across 11 cell types; (B) UMAP clustering results visualized by grouping based on SOCS3 expression in neural stem cells, illustrating cell aggregation and distribution, with each color representing a distinct cluster.

**
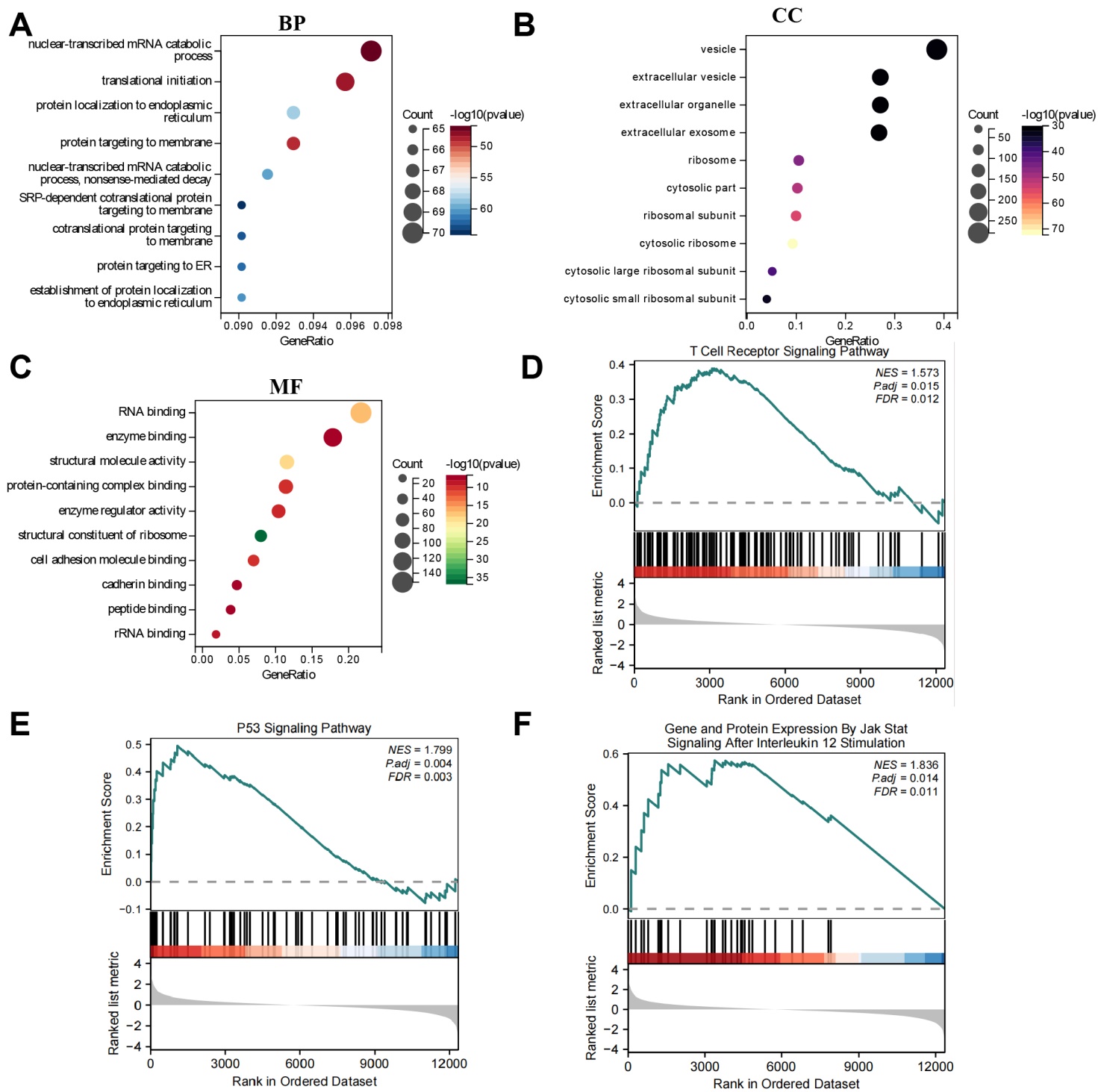
**

**Figure S3. GO enrichment analysis and GSEA enrichment plots of DEGs**

Note: BP (A), CC (B), MF (C); Detailed GSEA plots for T cell receptor signaling pathway (D), p53 signaling pathway (E), and Jak-STAT signaling pathway (F).

**
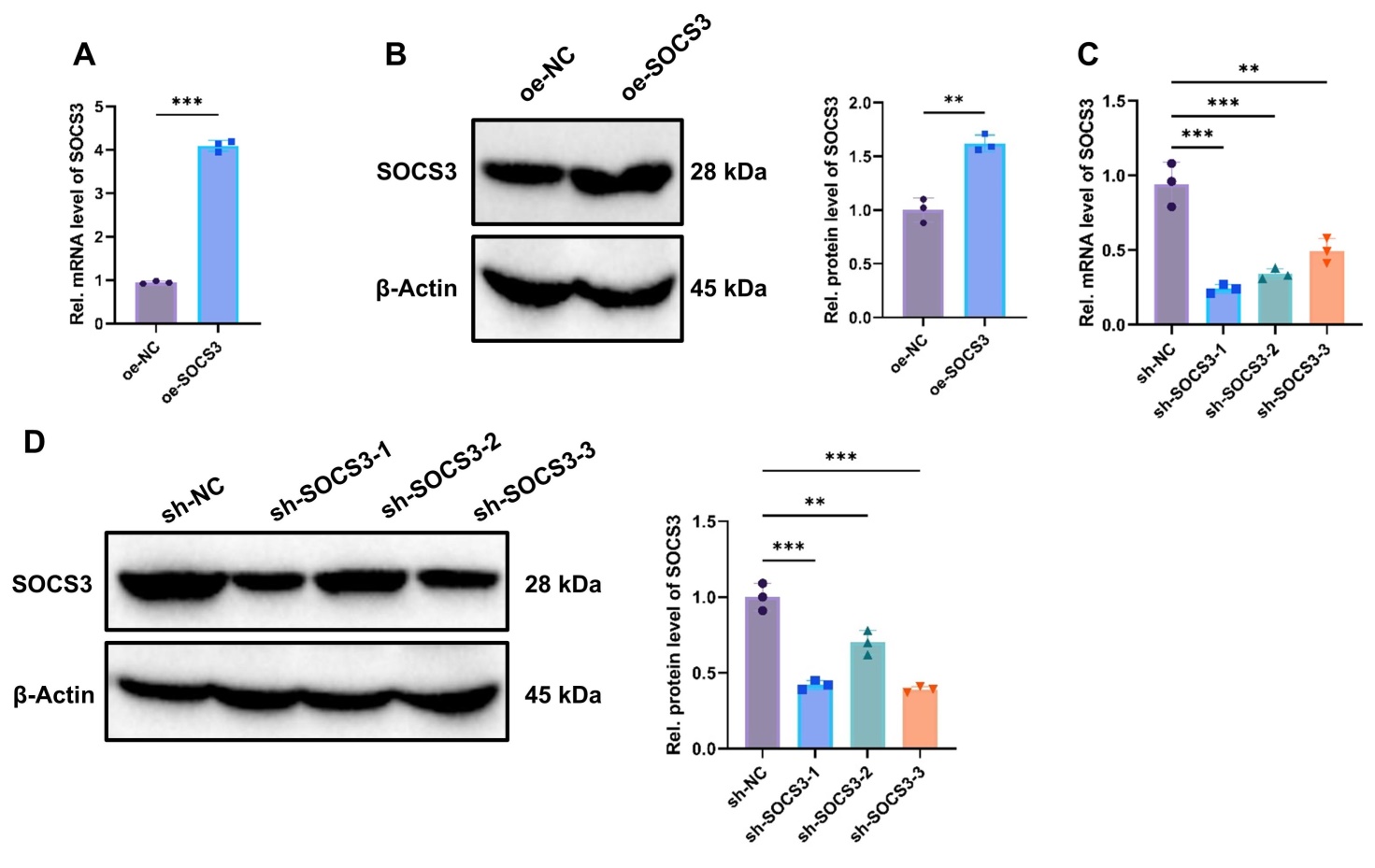
**

**Figure S4. Verification of SOCS3 Overexpression and Knockdown Efficiency.**

Note: (A-B) RT-qPCR and WB analysis of SOCS3 overexpression efficiency; (C-D) RT-qPCR and WB analysis of SOCS3 knockdown efficiency. ** indicates comparison between two groups, *p* < 0.01; *** *p* < 0.001. Cell experiments were repeated three times.

**
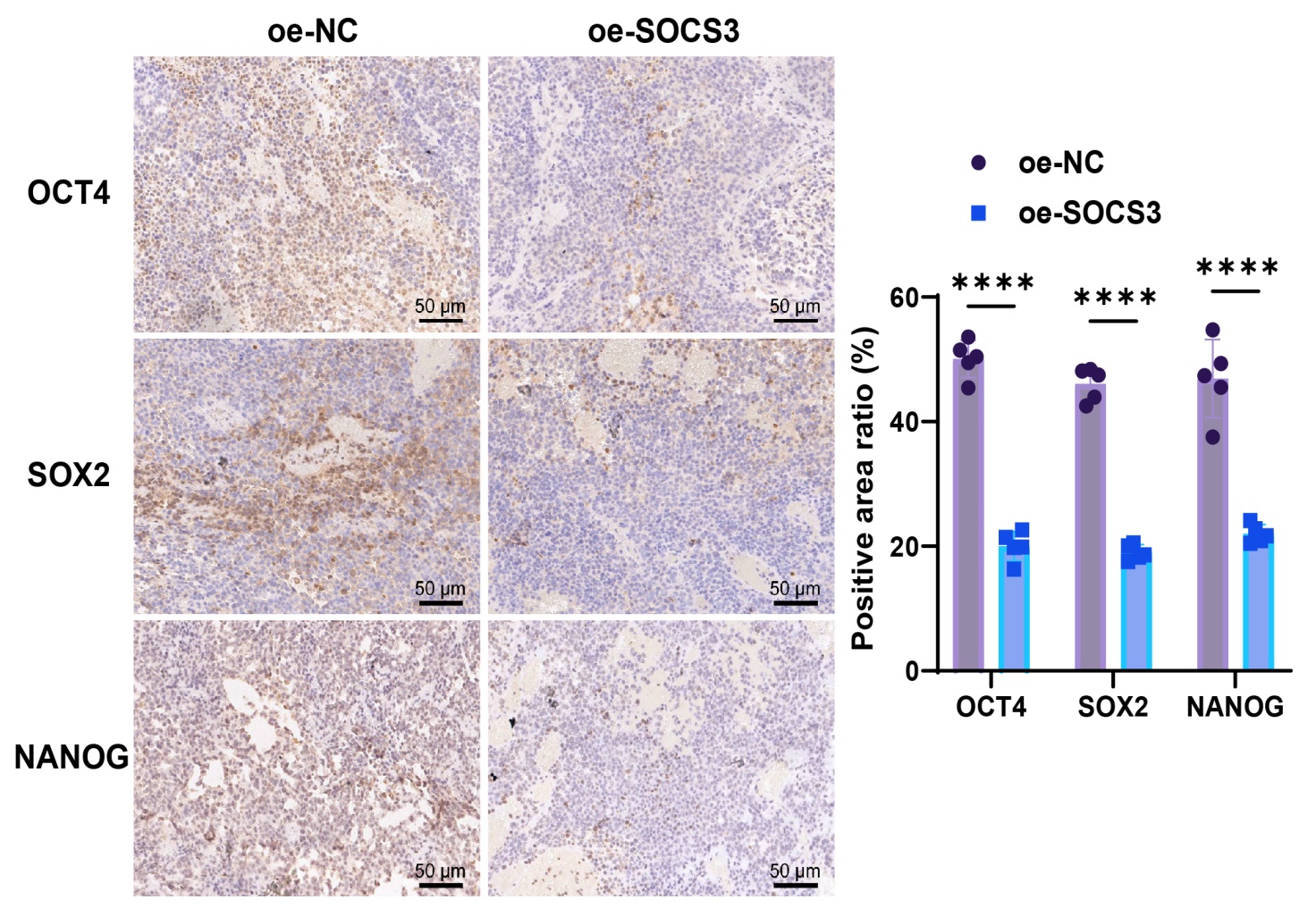
**

**Figure S5. SOCS3 overexpression significantly reduces stemness marker expression in the in vivo model.**

Note: Immunohistochemical detection of stemness markers in xenograft tumors and corresponding quantification results.

**Table S1. Primary Antibody Manufacturer Information.**

| **Antibody Name** | **Manufacturer** | **Antibody Item Number** | **Concentration** |
| --- | --- | --- | --- |
| SOCS3 | ABCAM | ab280884 | 1:1000 |
| Ki67 | ABCAM | ab16667 | 1:500 |
| PCNA | CST | #13110 | 1:1000 |
| Bax | CST | #2772 | 1:1000 |
| Caspase-3 | CST | #9662 | 1:1000 |
| Bcl-2 | CST | #15071 | 1:1000 |
| β-Actin | CST | #4970 | 1:1000 |
| STAT3 | CST | #12640 | 1:1000 |
| p-STAT3 | CST | #4113 | WB: 1:2000; IF: 1:100 |
| OCT4 | CST | #75463 | WB: 1:1000; IF: 1:200; IHC: 1:200 |
| SOX2 | CST | #23064 | WB: 1:1000; IF: 1:400; IHC: 1:200 |
| NANOG | CST | #4903 | WB: 1:2000 |
| NANOG | Thermo fisher | PA5-18406 | IF: 1:800; IHC: 1:200 |
| IgG H&L (Alexa Fluor® 488) | ABCAM | ab150113 | 1:400 |
| IgG H&L (Alexa Fluor® 647) | ABCAM | ab150075 | 1:200 |
| IgG H&L (Alkaline Phosphatase) | ABCAM | ab6886 | 1:200 |

**Table S2. RT-qPCR Primer Sequences.**

| **Gene** | **Forward Primer (5′-3′)** |
| --- | --- |
| SOCS3-F | CATCTCTGTCGGAAGACCGTCA |
| SOCS3-R | GCATCGTACTGGTCCAGGAACT |
| Ki67-F | GAAAGAGTGGCAACCTGCCTTC |
| Ki67-R | GCACCAAGTTTTACTACATCTGCC |
| PCNA-F | CCTGCTGGGATATTAGCTCCA |
| PCNA-R | CAGCGGTAGGTGTCGAAGC |
| β-actin-F | GGCACCCAGCACAATGAAG |
| β-actin-R | CCGATCCACACGGAGTACTTG |

**Table S3 Proportions of each cell type in SOCS3-High and SOCS3-Low groups.**

| **cell type** | **SOCS3-High** | **SOCS3-Low** |
| --- | --- | --- |
| Astrocytes | 0.202906977 | 0.300695905 |
| OPCs | 0.070930233 | 0.118789448 |
| T cells | 0.122674419 | 0.095403787 |
| Microglias | 0.31744186 | 0.218643793 |
| Neurons | 0.099418605 | 0.087150024 |
| Mesenchymal cells | 0.077325581 | 0.055834277 |
| Neural progenitor | 0.036627907 | 0.059151966 |
| Dendritic cells | 0.044186047 | 0.036818255 |
| NK cells | 0.013372093 | 0.013372093 |
| Oligodendrocytes | 0.009883721 | 0.006554459 |
| B cells | 0.005232558 | 0.007120893 |

**Table S4 Differentially expressed genes (DEGs) between SOCS3-Low and SOCS3-High glioma stem cells.**

| **Gene** | avg_log2FC | p_val |
| --- | --- | --- |
| TSTD1 | 0.93975804 | 2.85E-97 |
| AC083805.1 | 1.446996983 | 3.32E-94 |
| EEF1AKMT3 | 0.818585646 | 1.20E-60 |
| RPL39L | 1.29298777 | 1.63E-46 |
| ATP23 | 2.093169132 | 2.42E-40 |
| ADAM12 | 0.546408754 | 6.91E-40 |
| AVIL | 1.133273012 | 2.40E-34 |
| SHTN1 | 0.998222998 | 1.05E-33 |
| AGAP2 | 1.368258679 | 9.11E-33 |
| XIST | 1.193092999 | 4.72E-31 |
| METTL1 | 1.718423255 | 1.13E-29 |
| RPL11 | 1.701561788 | 2.23E-28 |
| CDKN2A | 2.822741718 | 3.12E-27 |
| RPS8 | 1.371196023 | 1.33E-26 |
| RPL22 | 1.327778041 | 5.13E-26 |
| COL1A2 | 0.947601216 | 5.86E-26 |
| RPS6 | 1.189520208 | 6.13E-26 |
| RPLP1 | 1.192475616 | 1.53E-25 |
| FXYD5 | 0.566843707 | 1.04E-24 |
| TSPAN31 | 2.340657518 | 1.17E-24 |
| OXA1L | 1.614351967 | 1.83E-24 |
| RPL12 | 1.221813491 | 3.24E-23 |
| RPL28 | 0.946827457 | 4.21E-23 |
| FABP5 | 2.588246841 | 6.39E-23 |
| RPS17 | 1.04418104 | 6.62E-22 |
| ARHGAP24 | 0.737013906 | 3.44E-21 |
| IFITM3 | 0.684724362 | 3.69E-21 |
| MYO1F | 0.51245891 | 5.34E-21 |
| RPL36A | 0.999694796 | 6.34E-21 |
| FGL2 | 0.774193162 | 1.36E-20 |
| SNHG25 | 0.974431153 | 3.48E-20 |
| RPS19 | 0.868380261 | 5.53E-20 |
| IGFBP5 | -3.033678089 | 7.95E-20 |
| MT1E | 0.599267087 | 1.17E-19 |
| LDHB | -1.134769792 | 1.34E-19 |
| PTMS | -1.207875664 | 1.43E-19 |
| GAPDH | -1.033271471 | 1.54E-19 |
| RPS2 | 1.063441998 | 2.04E-19 |
| RPL22L1 | -1.783909791 | 3.30E-19 |
| KLF4 | 0.536188779 | 4.13E-19 |
| SCG2 | 0.517358151 | 4.25E-19 |
| RPL37A | 0.89880782 | 7.75E-19 |
| CD4 | 0.569377502 | 8.43E-19 |
| HLA-DQA1 | 1.323602392 | 8.92E-19 |
| FIP1L1 | -2.33815207 | 1.88E-18 |
| RPL18A | 0.921049309 | 2.03E-18 |
| TSFM | 3.051291354 | 2.25E-18 |
| RPS4X | 1.139481103 | 6.42E-18 |
| RPS24 | 0.716644055 | 6.96E-18 |
| RPS14 | 0.780689191 | 1.10E-17 |
| SERPINB1 | 0.641065804 | 1.98E-17 |
| FPR1 | 0.619781885 | 2.98E-17 |
| RPL13A | 0.737416629 | 3.39E-17 |
| RPL5 | 0.915261307 | 4.10E-17 |
| CBX5 | -1.295637669 | 4.22E-17 |
| EVI2B | 0.632210488 | 4.44E-17 |
| NRXN1 | -1.49824643 | 6.34E-17 |
| RPL37 | 0.920180981 | 7.17E-17 |
| CX3CR1 | 0.96928822 | 8.91E-17 |
| RPL13 | 0.746922892 | 9.31E-17 |
| SERPINA1 | 0.73861865 | 1.06E-16 |
| RPL39 | 0.707070245 | 1.07E-16 |
| EPN2 | -1.608167643 | 1.09E-16 |
| DSTN | -1.091469633 | 2.00E-16 |
| RPS10 | 0.745515963 | 2.34E-16 |
| RPS26 | -0.654989199 | 2.56E-16 |
| NOVA1 | -1.445428841 | 2.63E-16 |
| RPS16 | 0.839629852 | 3.05E-16 |
| AC084033.3 | 2.88066059 | 3.21E-16 |
| RPS27 | 0.97719323 | 3.36E-16 |
| DOCK8 | 0.57283424 | 4.66E-16 |
| UBA52 | 0.698331354 | 5.41E-16 |
| RPS23 | 0.678296976 | 6.71E-16 |
| C1QL1 | -1.679246441 | 7.96E-16 |
| FCGR1A | 1.226040349 | 8.07E-16 |
| SKAP2 | 0.76359513 | 8.12E-16 |
| FTL | 1.339868979 | 8.79E-16 |
| RPS4Y1 | -1.595943058 | 1.06E-15 |
| IFITM2 | 0.56549688 | 1.49E-15 |
| RPS3A | 0.724520878 | 1.71E-15 |
| RPS11 | 0.683511822 | 1.74E-15 |
| CD9 | -1.706916426 | 1.92E-15 |
| RPL30 | 0.716336665 | 2.71E-15 |
| NCKAP1L | 0.513154125 | 3.22E-15 |
| DENND3 | 0.535364862 | 3.72E-15 |
| CLEC7A | 0.742948965 | 3.90E-15 |
| C3AR1 | 0.883346654 | 3.92E-15 |
| PHLDA1 | -1.499192268 | 4.06E-15 |
| CD14 | 1.464888761 | 4.48E-15 |
| CSF1R | 1.310941291 | 4.86E-15 |
| MEF2C | 1.06652067 | 6.36E-15 |
| S100B | -1.381328178 | 7.02E-15 |
| CD53 | 0.704194962 | 8.76E-15 |
| ATP5IF1 | 0.789410352 | 1.85E-14 |
| IGSF6 | 0.807076146 | 2.21E-14 |
| RPL4 | 0.803955752 | 2.23E-14 |
| SLC2A5 | 0.856040433 | 2.25E-14 |
| 9-Mar | 2.916869621 | 2.35E-14 |
| RPL24 | 0.683342138 | 2.37E-14 |
| PABPC4 | 0.690884896 | 2.46E-14 |
| RPS21 | 0.873496081 | 2.49E-14 |
| APBB1IP | 0.910911162 | 2.50E-14 |
| KLF2 | 1.101171304 | 3.38E-14 |
| CAPG | 0.565412823 | 3.90E-14 |
| VAMP8 | 0.865079046 | 4.22E-14 |
| RPS28 | 0.667028827 | 4.54E-14 |
| RPS20 | 0.651593281 | 4.68E-14 |
| OLIG1 | -1.304275424 | 6.04E-14 |
| NCK2 | 0.57334633 | 6.33E-14 |
| CD84 | 0.591288768 | 6.92E-14 |
| SLCO2B1 | 0.805244849 | 7.28E-14 |
| LIMA1 | -1.288988573 | 7.38E-14 |
| RGS1 | 1.196129307 | 8.01E-14 |
| YWHAE | -0.837736419 | 8.47E-14 |
| FYN | -1.14973735 | 9.70E-14 |
| TUBA1C | 0.564934714 | 1.05E-13 |
| RPL14 | 0.652189514 | 1.07E-13 |
| HIST3H2A | 0.709748961 | 1.50E-13 |
| SLC11A1 | 0.835101287 | 1.55E-13 |
| OLFM1 | -1.4470145 | 1.57E-13 |
| CYBB | 1.346222283 | 1.84E-13 |
| RTN1 | -1.495752469 | 1.87E-13 |
| TBXAS1 | 0.640549668 | 1.95E-13 |
| RPS15A | 0.608486006 | 1.98E-13 |
| CDC42 | 0.749068709 | 2.04E-13 |
| CD63 | -1.089793175 | 2.07E-13 |
| TNC | 0.898573921 | 2.32E-13 |
| HLA-DQB1 | 1.233188759 | 2.54E-13 |
| EIF3I | 0.981184125 | 2.59E-13 |
| SSU72 | 0.76933123 | 3.08E-13 |
| TSC22D1 | -2.193121573 | 3.21E-13 |
| GPR34 | 1.153648194 | 3.22E-13 |
| PTP4A3 | 0.616089553 | 3.52E-13 |
| FOS | 1.963547536 | 3.73E-13 |
| BCAN | -1.103314614 | 3.82E-13 |
| SERBP1 | 1.004105951 | 3.86E-13 |
| BEX3 | -1.054296912 | 3.87E-13 |
| HCST | 0.785239351 | 4.61E-13 |
| FCGR3A | 1.733067242 | 4.79E-13 |
| RPL10 | 0.661697995 | 4.82E-13 |
| KLF6 | 1.628497992 | 5.23E-13 |
| RPS3 | 0.671195067 | 5.93E-13 |
| ITGB2 | 0.973820449 | 6.95E-13 |
| RPS18 | 0.897278364 | 7.37E-13 |
| MDK | -1.197019814 | 7.41E-13 |
| BMP7 | -1.074203643 | 7.48E-13 |
| RPLP2 | 0.654143061 | 7.70E-13 |
| PLD4 | 0.685551608 | 8.00E-13 |
| RAMP2 | -1.083538116 | 8.13E-13 |
| KLHDC8A | 0.50434413 | 9.27E-13 |
| APOD | -1.279179665 | 9.33E-13 |
| MLF2 | -1.044904969 | 9.55E-13 |
| NCAM1 | -1.169918696 | 1.02E-12 |
| PCSK1N | -1.17538745 | 1.13E-12 |
| CDK4 | 3.828265145 | 1.15E-12 |
| RPL18 | 0.670083198 | 1.16E-12 |
| RPL34 | 0.65478053 | 1.17E-12 |
| YBX1 | 1.233299936 | 1.44E-12 |
| DBI | -0.980461902 | 1.45E-12 |
| RPL36 | 0.90824583 | 1.55E-12 |
| PTPRC | 1.197527604 | 1.68E-12 |
| PLXDC2 | 0.935592459 | 1.91E-12 |
| REV3L | -1.326512503 | 2.62E-12 |
| GNG5 | 0.758022051 | 2.63E-12 |
| VSIR | 0.819101024 | 2.68E-12 |
| HLA-DMB | 0.971631185 | 2.72E-12 |
| WASF2 | 0.603793114 | 2.76E-12 |
| SLC38A1 | -1.14546597 | 2.76E-12 |
| RPL35 | 0.701349872 | 3.60E-12 |
| NRDC | 0.578490596 | 3.94E-12 |
| MS4A6A | 0.849025186 | 5.23E-12 |
| RPSA | 0.707595853 | 5.41E-12 |
| JUN | 1.342125797 | 5.96E-12 |
| SRGN | 1.363095522 | 6.20E-12 |
| FCER1G | 1.077440101 | 6.43E-12 |
| RPL7A | 0.617030944 | 7.17E-12 |
| USP46 | -2.081071061 | 7.47E-12 |
| ATP6V0B | 0.68834898 | 8.07E-12 |
| SCG3 | -1.41806291 | 8.87E-12 |
| RAB31 | -1.030729073 | 8.89E-12 |
| RPL32 | 0.656482619 | 9.61E-12 |
| LPAR6 | 0.505283508 | 1.14E-11 |
| RPL29 | 0.734481058 | 1.28E-11 |
| OLR1 | 0.660342231 | 1.31E-11 |
| PABPC1 | 0.754147838 | 1.74E-11 |
| CAPZA1 | 0.507597005 | 1.92E-11 |
| ZFP36 | 1.357803157 | 2.27E-11 |
| C3 | 2.160689559 | 2.97E-11 |
| ALOX5AP | 1.217171019 | 3.20E-11 |
| BASP1 | -0.856537057 | 3.24E-11 |
| SCRG1 | -1.019740691 | 3.45E-11 |
| DYNLT1 | -0.955421285 | 4.07E-11 |
| C1QC | 2.171486361 | 4.28E-11 |
| TMEM158 | 0.590951543 | 4.74E-11 |
| PTPRZ1 | -0.944759104 | 5.64E-11 |
| TREM2 | 0.907760742 | 5.86E-11 |
| GNL2 | 0.501475435 | 5.97E-11 |
| TCF12 | 0.535335933 | 6.21E-11 |
| RPL10A | 0.530418013 | 6.35E-11 |
| PEBP1 | -0.731916446 | 7.28E-11 |
| ARPC1B | 0.91799807 | 7.38E-11 |
| C1QA | 2.080426752 | 7.84E-11 |
| RPS7 | 0.667789093 | 9.28E-11 |
| SPI1 | 0.603018407 | 9.59E-11 |
| PHC2 | 0.52545716 | 9.87E-11 |
| BST2 | 0.620549665 | 1.16E-10 |
| HLA-DMA | 1.120231696 | 1.19E-10 |
| ZCRB1 | -0.961560745 | 1.24E-10 |
| NFIB | -1.034853639 | 1.35E-10 |
| UBB | -0.692993683 | 1.37E-10 |
| SRSF4 | 0.61421437 | 1.38E-10 |
| SPARC | -0.710568822 | 1.39E-10 |
| GSTP1 | -0.740429467 | 1.65E-10 |
| HIST1H4C | -1.399801603 | 1.71E-10 |
| RPL27A | 0.556229026 | 1.73E-10 |
| EEF1B2 | 0.671695495 | 1.86E-10 |
| AIF1 | 1.918904028 | 1.87E-10 |
| AKR1A1 | 0.594743434 | 2.02E-10 |
| S100A10 | 0.572130128 | 2.11E-10 |
| MCL1 | 0.889080996 | 2.11E-10 |
| OTUD1 | 0.692528828 | 2.15E-10 |
| RPL8 | 0.661283219 | 2.28E-10 |
| LPCAT2 | 0.832742863 | 2.41E-10 |
| BLOC1S1 | -0.874088929 | 2.53E-10 |
| TM4SF1 | -1.395564256 | 2.70E-10 |
| CTSH | 0.592230233 | 3.16E-10 |
| MINOS1 | 0.720648945 | 3.23E-10 |
| MRPL51 | -0.78498749 | 3.33E-10 |
| RPL17 | 0.790411862 | 3.34E-10 |
| HLA-DRB1 | 1.706787576 | 3.51E-10 |
| PFDN5 | -0.660203904 | 3.64E-10 |
| CTSC | 0.662041049 | 3.69E-10 |
| RPS15 | 0.68248774 | 3.96E-10 |
| RPL15 | 0.569674274 | 4.00E-10 |
| C1QB | 2.177682642 | 4.08E-10 |
| PSMB4 | 0.859383033 | 4.26E-10 |
| VIM | 1.506485594 | 5.09E-10 |
| RAMP1 | -1.044500986 | 5.47E-10 |
| ENO1 | 1.115026883 | 5.54E-10 |
| PTN | -0.794150989 | 6.01E-10 |
| SCFD2 | -1.663158737 | 6.29E-10 |
| NAP1L1 | -0.689431104 | 6.33E-10 |
| FXYD6 | -0.876071486 | 6.64E-10 |
| RPS13 | 0.574506595 | 6.69E-10 |
| PTGES3 | -0.724773817 | 7.07E-10 |
| TMEM97 | -1.020448398 | 7.24E-10 |
| GPM6B | -0.793805082 | 8.43E-10 |
| PGRMC1 | -0.967788151 | 9.27E-10 |
| PLP1 | -1.101226958 | 1.19E-09 |
| TDG | 1.301645488 | 1.25E-09 |
| TTC3 | -0.768470558 | 1.26E-09 |
| CAP1 | 0.507632469 | 1.30E-09 |
| RGS2 | 0.541127162 | 1.35E-09 |
| CYBA | 1.62615076 | 1.39E-09 |
| CA10 | -0.863304928 | 1.45E-09 |
| RPLP0 | 0.608200185 | 1.50E-09 |
| UTP11 | 0.557137676 | 1.52E-09 |
| CPE | -1.023110313 | 1.68E-09 |
| HES1 | -1.157718546 | 1.76E-09 |
| CTSS | 0.949193894 | 1.76E-09 |
| TYROBP | 1.360619549 | 1.77E-09 |
| OCIAD2 | 1.18024394 | 1.82E-09 |
| MRPL20 | 0.759349321 | 2.01E-09 |
| SH3BGRL3 | 0.762125999 | 2.44E-09 |
| ATP5MC2 | -0.62022329 | 2.54E-09 |
| STRAP | -0.843749822 | 2.64E-09 |
| CEBPB | 0.832643453 | 2.81E-09 |
| RGS10 | 0.906436141 | 2.98E-09 |
| MS4A7 | 0.566964161 | 3.36E-09 |
| A2M | 1.451149162 | 3.65E-09 |
| GABBR1 | -0.828891108 | 4.69E-09 |
| EEF2 | 0.509548385 | 5.02E-09 |
| SRRM1 | 0.608575347 | 5.13E-09 |
| SSH2 | 0.548167965 | 5.67E-09 |
| PRDX1 | 0.848623571 | 6.01E-09 |
| TMSB4X | 1.272723741 | 6.23E-09 |
| ATP5F1B | -0.843638803 | 6.34E-09 |
| EEF1A1 | 0.515009502 | 6.47E-09 |
| POLR2J3.1 | -1.197226676 | 6.56E-09 |
| MYL6 | -0.588977887 | 6.66E-09 |
| RPL31 | 0.654656934 | 6.73E-09 |
| C4orf48 | -0.76138622 | 6.87E-09 |
| CNTN1 | -1.291481522 | 8.01E-09 |
| CHIC2 | -1.46697128 | 8.06E-09 |
| SELENOW | -0.759616699 | 8.41E-09 |
| TMEM98 | -0.719563555 | 8.58E-09 |
| TOP2A | -1.615544348 | 9.70E-09 |
| CENPV | -0.744486784 | 1.01E-08 |
| RPL3 | 0.543167004 | 1.03E-08 |
| TUBB2B | -0.807142804 | 1.07E-08 |
| GRIA3 | -0.83862233 | 1.10E-08 |
| FAT3 | -1.037522435 | 1.14E-08 |
| KRAS | -0.896116841 | 1.14E-08 |
| CEBPD | 1.327309994 | 1.17E-08 |
| UFC1 | 0.670031561 | 1.22E-08 |
| CNPY2 | -0.861778036 | 1.49E-08 |
| MIA | -1.248176306 | 1.50E-08 |
| PCDH17 | -0.955470152 | 1.58E-08 |
| DEK | -0.801437389 | 1.66E-08 |
| MRPL18 | -0.928064701 | 1.77E-08 |
| EBNA1BP2 | 0.58300402 | 1.86E-08 |
| RPL38 | 0.582470933 | 1.90E-08 |
| BEX1 | -0.929193877 | 2.04E-08 |
| CLIC1 | 0.610241775 | 2.10E-08 |
| RPS5 | 0.709093134 | 2.22E-08 |
| LNX1 | -1.078297145 | 2.79E-08 |
| LAPTM5 | 1.215551735 | 2.83E-08 |
| SEC11C | -0.919819443 | 2.88E-08 |
| PSMB2 | 0.625756492 | 2.90E-08 |
| RACK1 | 0.655713409 | 3.40E-08 |
| PCDH9 | -1.399299036 | 3.42E-08 |
| CLU | -0.66768905 | 3.45E-08 |
| HLA-DRA | 2.233182427 | 3.65E-08 |
| RPS25 | 0.513107743 | 3.86E-08 |
| ACAT2 | -1.257777168 | 4.19E-08 |
| NAA38 | -0.770603741 | 5.24E-08 |
| FAIM2 | -0.71440543 | 5.48E-08 |
| YBX3 | -0.791377813 | 5.78E-08 |
| FGD4 | 0.512124555 | 6.45E-08 |
| BTG2 | 1.049297465 | 6.82E-08 |
| GPM6A | -0.831513365 | 6.92E-08 |
| OSBPL8 | -0.851429656 | 7.00E-08 |
| ST8SIA1 | -0.784390038 | 7.25E-08 |
| CD24 | -0.930877536 | 7.25E-08 |
| NASP | 0.767182596 | 7.41E-08 |
| NDRG2 | -0.996696207 | 7.65E-08 |
| EIF1AY | -0.75847289 | 8.62E-08 |
| PRDX5 | -0.65517258 | 9.89E-08 |
| TMEM59 | 0.516163411 | 1.06E-07 |
| KCTD12 | 1.09797103 | 1.08E-07 |
| TSC22D4 | -0.871312332 | 1.08E-07 |
| TIMP2 | -0.831423046 | 1.12E-07 |
| COTL1 | -1.01782468 | 1.19E-07 |
| PHGDH | -0.960719585 | 1.20E-07 |
| CORO1A | 0.501958252 | 1.20E-07 |
| NPC2 | 1.829142382 | 1.24E-07 |
| TCP1 | -0.814405245 | 1.27E-07 |
| ZEB1 | -0.973198383 | 1.27E-07 |
| FEZ1 | -0.76871795 | 1.61E-07 |
| FOSB | 0.646573631 | 1.69E-07 |
| SRSF5 | 0.594231628 | 1.71E-07 |
| DUSP1 | 1.445368728 | 1.79E-07 |
| CD74 | 2.354176451 | 1.82E-07 |
| TXNDC17 | -0.756412425 | 1.83E-07 |
| ATP6V0E2 | -0.770869343 | 1.91E-07 |
| UBE2J2 | 0.526655791 | 2.13E-07 |
| COA3 | -0.801656611 | 2.27E-07 |
| PA2G4 | -0.626766557 | 2.39E-07 |
| PARK7 | 0.718443922 | 2.40E-07 |
| FAM213A | -0.718458506 | 2.47E-07 |
| RNF157 | -0.766421361 | 2.56E-07 |
| HOXC9 | -0.670031015 | 2.63E-07 |
| AHI1 | -0.870580503 | 2.64E-07 |
| TRIM9 | -0.875270613 | 3.04E-07 |
| SYF2 | 0.625214115 | 3.07E-07 |
| SOBP | -0.744925875 | 3.30E-07 |
| DST | -0.884790143 | 3.57E-07 |
| ARHGDIB | 0.808901166 | 3.61E-07 |
| MAP2 | -0.924865395 | 3.63E-07 |
| HOXC8 | -0.556765026 | 3.89E-07 |
| SMC2 | -0.776135241 | 3.97E-07 |
| NDUFC2 | -0.526628704 | 4.17E-07 |
| KLRC2 | -0.998463891 | 4.30E-07 |
| KCNQ1OT1 | -1.131416611 | 4.32E-07 |
| FGFR1 | -0.951785585 | 4.35E-07 |
| NTRK2 | -1.022714061 | 4.45E-07 |
| SRM | 0.665955985 | 4.47E-07 |
| THSD7A | -0.625002479 | 4.55E-07 |
| CKB | 0.933220048 | 4.63E-07 |
| MYH10 | -0.819076034 | 4.75E-07 |
| CYB5R3 | -0.890479774 | 4.89E-07 |
| NPY | -2.30686129 | 5.43E-07 |
| SCD5 | -0.88582952 | 6.04E-07 |
| SLC22A17 | -0.791808943 | 6.07E-07 |
| TCEAL9 | -0.898339889 | 6.11E-07 |
| NFKBIA | 0.612729972 | 6.35E-07 |
| LSAMP | -0.766083471 | 6.46E-07 |
| KRT10 | -0.726620142 | 6.65E-07 |
| TCF4 | -0.700210093 | 6.79E-07 |
| UQCRH | 0.677337982 | 7.37E-07 |
| ARL4C | 0.831588096 | 7.79E-07 |
| CSRP2 | -0.692302641 | 7.80E-07 |
| FABP6 | -0.715421929 | 7.81E-07 |
| TMBIM6 | -0.610864672 | 8.24E-07 |
| RNASEH2C | -0.829744015 | 8.51E-07 |
| SLITRK4 | -0.580367932 | 9.10E-07 |
| SERPINE2 | -1.019649637 | 9.69E-07 |
| CCND2 | -0.859445265 | 1.08E-06 |
| BCAT1 | -0.743939432 | 1.11E-06 |
| LMO4 | -0.960953176 | 1.22E-06 |
| DDX3X | 0.607188314 | 1.31E-06 |
| JPH4 | -0.638230481 | 1.31E-06 |
| CAND1 | -0.735377519 | 1.36E-06 |
| SNRPE | 0.70414784 | 1.38E-06 |
| ERGIC2 | -0.750157242 | 1.38E-06 |
| CALM3 | -0.706829518 | 1.43E-06 |
| ZNF462 | -0.838718123 | 1.45E-06 |
| LINC01896 | -0.94744889 | 1.50E-06 |
| SORL1 | 1.124507559 | 1.51E-06 |
| TSPO | 0.545926676 | 1.53E-06 |
| HOXA10 | -0.552580582 | 1.54E-06 |
| PRAME | -0.553447026 | 1.57E-06 |
| TMX4 | -0.859936348 | 1.88E-06 |
| S100A16 | -1.112796565 | 1.90E-06 |
| SPARCL1 | -0.6881304 | 2.12E-06 |
| USP22 | -0.755761028 | 2.20E-06 |
| OLIG2 | -0.795988306 | 2.21E-06 |
| SERPINB6 | -0.710190176 | 2.22E-06 |
| THY1 | -0.919680003 | 2.30E-06 |
| IFI16 | 0.800057678 | 2.39E-06 |
| APOC1 | 1.562497725 | 2.47E-06 |
| STMN1 | 0.933467608 | 2.54E-06 |
| CMTM5 | -0.624630643 | 2.54E-06 |
| AP2B1 | -0.701262578 | 2.62E-06 |
| DNAJC1 | -0.845189936 | 2.75E-06 |
| CAV1 | -0.729032958 | 2.80E-06 |
| GPATCH4 | 0.7194068 | 2.88E-06 |
| HTRA1 | -1.202851882 | 2.89E-06 |
| PLEKHH2 | -0.722453433 | 3.01E-06 |
| ZBTB20 | -0.770132443 | 3.10E-06 |
| SNX3 | -0.576784167 | 3.25E-06 |
| DNAJC8 | 0.509896983 | 3.26E-06 |
| NRXN2 | -0.641881729 | 3.30E-06 |
| NLRP1 | -0.71238161 | 3.34E-06 |
| CCDC88A | -0.652993186 | 3.77E-06 |
| ETS2 | 0.523746602 | 3.90E-06 |
| RABEP1 | -0.668985158 | 4.18E-06 |
| PLEKHB1 | -0.79264409 | 4.22E-06 |
| HLA-E | 0.974907031 | 4.26E-06 |
| SPCS2 | -0.651979634 | 4.26E-06 |
| VAMP2 | -0.782689985 | 4.26E-06 |
| TCEAL2 | -0.823447695 | 4.46E-06 |
| ITM2C | -0.989584117 | 4.47E-06 |
| TNR | -1.178839126 | 4.48E-06 |
| NGFR | -1.119688699 | 4.55E-06 |
| WNT7B | -0.537277433 | 4.67E-06 |
| EID1 | -0.669235537 | 4.68E-06 |
| HLA-DPA1 | 2.105269691 | 4.77E-06 |
| STUB1 | -0.608370224 | 4.88E-06 |
| NPDC1 | -0.743774542 | 4.90E-06 |
| PHF19 | 0.560110097 | 5.15E-06 |
| KRR1 | -0.736241635 | 5.16E-06 |
| SETD5 | -0.736649604 | 5.26E-06 |
| KMT2A | -0.771348231 | 5.56E-06 |
| SOD1 | -0.529879374 | 5.84E-06 |
| ARMCX3 | -0.797075475 | 6.58E-06 |
| ATP1B2 | -0.548120058 | 6.60E-06 |
| CADM2 | -0.820547215 | 6.68E-06 |
| USP54 | -0.520747392 | 7.17E-06 |
| PDE1C | -0.517882897 | 7.19E-06 |
| TCEAL4 | -0.711223771 | 7.60E-06 |
| LEF1 | -0.833191384 | 7.72E-06 |
| TTYH1 | -0.701104294 | 7.79E-06 |
| LST1 | 0.805579915 | 7.81E-06 |
| REL | 0.540906134 | 8.15E-06 |
| HEY1 | -0.771407806 | 8.56E-06 |
| MYL6B | -0.651514469 | 8.74E-06 |
| FAM181B | -0.827538918 | 8.75E-06 |
| S100A11 | 1.193660403 | 8.84E-06 |
| CCDC167 | -0.744180565 | 9.03E-06 |
| CXXC5 | -0.768437938 | 9.20E-06 |
| DCTN2 | -0.778591704 | 9.49E-06 |
| SYT1 | -0.52140659 | 9.81E-06 |
| SAMHD1 | 0.595902622 | 1.03E-05 |
| SLC1A2 | -0.610403124 | 1.06E-05 |
| NDUFS5 | 0.564748856 | 1.11E-05 |
| CXXC4 | -0.512250683 | 1.18E-05 |
| FHL1 | -0.728633438 | 1.23E-05 |
| TMEM106B | -0.652267388 | 1.24E-05 |
| SCG5 | -0.612611084 | 1.26E-05 |
| GLUL | 0.800291941 | 1.30E-05 |
| ANXA2 | 0.537766796 | 1.32E-05 |
| SALL3 | -0.733507044 | 1.35E-05 |
| CAV2 | -0.509528486 | 1.45E-05 |
| IFNGR1 | 1.315798203 | 1.51E-05 |
| CYFIP1 | 0.663575463 | 1.57E-05 |
| PMP22 | -0.621754766 | 1.71E-05 |
| AZGP1 | -0.923190229 | 1.71E-05 |
| SPTLC2 | 0.513222337 | 1.74E-05 |
| AC022075.1 | -0.595962987 | 1.75E-05 |
| CYCS | -0.548946578 | 1.83E-05 |
| SF3B5 | -0.584923286 | 1.85E-05 |
| FKBP2 | -0.702669687 | 1.90E-05 |
| GCSH | -0.672465732 | 1.98E-05 |
| TSHZ2 | -0.734243928 | 2.01E-05 |
| APLP2 | -0.603681902 | 2.03E-05 |
| SAT2 | -0.647473465 | 2.12E-05 |
| LYPD1 | -0.562399515 | 2.22E-05 |
| NDUFB10 | -0.563804844 | 2.24E-05 |
| ZDHHC14 | -0.653181375 | 2.27E-05 |
| CDKN3 | -1.052013667 | 2.29E-05 |
| SMARCC2 | -0.695841266 | 2.38E-05 |
| MGST1 | -0.523545781 | 2.41E-05 |
| MGLL | -0.797509533 | 2.55E-05 |
| CMBL | -0.602480398 | 2.73E-05 |
| SEMA5A | -0.805146127 | 2.76E-05 |
| LRP1 | -0.673150017 | 2.95E-05 |
| AC007952.4 | 1.31314304 | 3.13E-05 |
| RAD51AP1 | -0.719706609 | 3.17E-05 |
| PRRX1 | -0.589057496 | 3.19E-05 |
| CPNE2 | -0.757421892 | 3.20E-05 |
| ADD3 | -0.660130879 | 3.26E-05 |
| PHLDB1 | -0.523407927 | 3.27E-05 |
| LINC01116 | -0.632967062 | 3.43E-05 |
| PSD3 | -0.730711184 | 3.49E-05 |
| RDX | -0.584556439 | 3.52E-05 |
| CDK1 | -0.923831698 | 3.69E-05 |
| TSPAN18 | -0.787907791 | 3.75E-05 |
| ETFRF1 | -0.581440393 | 3.87E-05 |
| RAN | -0.523180044 | 3.90E-05 |
| POLR2J | -0.589148621 | 4.13E-05 |
| PSIP1 | -0.583711677 | 4.13E-05 |
| SEC63 | -0.65074299 | 4.14E-05 |
| DCX | -0.738078408 | 4.18E-05 |
| PFN2 | -0.644422529 | 4.40E-05 |
| MAGEH1 | -0.678322744 | 4.41E-05 |
| ECI2 | -0.724148841 | 4.46E-05 |
| HMGB1 | -0.644453545 | 4.54E-05 |
| PGM3 | -0.559225468 | 4.70E-05 |
| RASSF8-AS1 | -0.528801117 | 4.73E-05 |
| YAF2 | -0.66500791 | 4.98E-05 |
| GRID2 | -0.607125056 | 5.12E-05 |
| ABI2 | -0.630238767 | 5.30E-05 |
| FRRS1L | -0.512852887 | 5.41E-05 |
| CSPG5 | -0.744509929 | 5.49E-05 |
| RPAIN | -0.640383176 | 5.64E-05 |
| VEGFB | -0.66715454 | 6.07E-05 |
| NUCB2 | -0.679085519 | 6.16E-05 |
| RBP1 | -1.479588075 | 6.30E-05 |
| DDX5 | 0.567208423 | 6.49E-05 |
| MRPS35 | -0.55917482 | 6.73E-05 |
| PIMREG | -0.886075031 | 7.13E-05 |
| TIMM8B | -0.58733 | 7.41E-05 |
| NCOR1 | -0.657451606 | 7.51E-05 |
| NTM | -0.586438103 | 7.81E-05 |
| JUNB | 0.944128698 | 8.07E-05 |
| BMP2K | 0.54778175 | 8.50E-05 |
| HMGN2 | 0.72287754 | 8.79E-05 |
| CENPK | -0.677616453 | 9.78E-05 |
| DDR1 | -0.52605848 | 9.79E-05 |
| TMEM160 | -0.524997255 | 0.000103965 |
| ST8SIA5 | -0.784390471 | 0.000106235 |
| SOX5 | -0.737870747 | 0.000107282 |
| PAK3 | -0.54486276 | 0.000107956 |
| OGFRL1 | 0.588626905 | 0.000112436 |
| ATXN7L3B | -0.650573373 | 0.000113937 |
| TPI1 | -0.536339002 | 0.000114858 |
| GTF2H5 | -0.622100192 | 0.00011804 |
| COX14 | -0.662444778 | 0.000126978 |
| NDUFA9 | -0.616514254 | 0.000129467 |
| MT3 | -0.622809142 | 0.000130613 |
| SLC35F1 | -0.592595 | 0.000132712 |
| C12orf10 | -0.582742554 | 0.000133764 |
| C11orf96 | -0.664933185 | 0.000135385 |
| WBP11 | -0.602008886 | 0.000135589 |
| ID2 | 0.530051528 | 0.000139889 |
| TNNT1 | -0.524282596 | 0.000141726 |
| HACD3 | -0.709921345 | 0.000144301 |
| APP | -0.704239989 | 0.000154302 |
| PPHLN1 | -0.673200066 | 0.000155509 |
| SOX2 | -0.68035625 | 0.000162061 |
| ITGAE | -0.670085789 | 0.000165946 |
| GNG2 | -0.66326815 | 0.000166403 |
| PPP1R12A | -0.658091825 | 0.000167298 |
| FAM89A | -0.626653788 | 0.00016743 |
| PSMB6 | -0.548004332 | 0.0001705 |
| EEF1A2 | 0.621201565 | 0.000171762 |
| PAFAH1B1 | -0.536335595 | 0.000181049 |
| THRA | -0.504713659 | 0.000183246 |
| YIPF3 | -0.63019194 | 0.000186294 |
| FKBP3 | -0.592072921 | 0.000187265 |
| FAM114A1 | -0.511971978 | 0.000197209 |
| COPRS | -0.576940733 | 0.000204563 |
| RBPJ | -0.557396264 | 0.000205761 |
| SEL1L3 | -0.585420499 | 0.00020819 |
| CKAP2 | -0.980299422 | 0.000209877 |
| PCDH7 | -0.671499116 | 0.000220865 |
| LRRC4C | -0.610414655 | 0.000222599 |
| BUB3 | -0.662366286 | 0.000226358 |
| NF1 | -0.538797436 | 0.000236243 |
| MBOAT2 | -0.590264853 | 0.000237595 |
| TCEAL5 | -0.658940887 | 0.000238827 |
| PRTFDC1 | -0.587124753 | 0.000239021 |
| FIBP | -0.586591629 | 0.000239304 |
| PBK | -0.712217656 | 0.0002415 |
| DDAH2 | -0.802173438 | 0.000245524 |
| GMNN | -0.612505976 | 0.000255946 |
| COL9A1 | -0.636081966 | 0.000262475 |
| FBLN1 | -0.561326507 | 0.000270704 |
| NDUFV1 | -0.58226951 | 0.00027473 |
| NCAM2 | -0.525557053 | 0.000308074 |
| TNIK | -0.591150349 | 0.000313105 |
| ISG15 | 0.686117091 | 0.000322814 |
| DCHS1 | -0.540525481 | 0.000323677 |
| PNN | -0.557692871 | 0.000328275 |
| GADD45B | 0.533497808 | 0.000329054 |
| MAGI2 | -0.508377552 | 0.000335287 |
| DNM1L | -0.564324394 | 0.000336725 |
| TTC9B | -0.570331354 | 0.00035171 |
| FOXM1 | -0.700295908 | 0.000369816 |
| PEA15 | -0.530268179 | 0.000376769 |
| STMN2 | -0.687244214 | 0.000402848 |
| DSEL | -0.553860956 | 0.000432141 |
| C1orf21 | -0.655174519 | 0.000433363 |
| QSER1 | -0.637293522 | 0.000444901 |
| CHD6 | -0.576081491 | 0.000445081 |
| DTNA | -0.511404541 | 0.000449871 |
| COPS6 | -0.511941904 | 0.000450222 |
| POU2F2 | 0.615295748 | 0.000455742 |
| FADS1 | -0.584448321 | 0.000477247 |
| AURKAIP1 | 0.507479949 | 0.000483758 |
| ASPM | -0.975502634 | 0.000519371 |
| GPAA1 | -0.600174723 | 0.000529186 |
| SPP1 | 0.793983183 | 0.000531458 |
| ARID4A | -0.594088088 | 0.000541019 |
| LRP6 | -0.643845852 | 0.000561905 |
| PLPP3 | -0.501235835 | 0.000583573 |
| DNER | -0.571489621 | 0.000601269 |
| SUCLG1 | -0.591978717 | 0.000612798 |
| TSPAN7 | -0.598986609 | 0.000615291 |
| CETN2 | -0.626036403 | 0.00063282 |
| IDI1 | -0.668254449 | 0.000634954 |
| CHID1 | -0.574542194 | 0.000636213 |
| UQCC2 | -0.518762691 | 0.000639185 |
| TJP1 | -0.587205807 | 0.000646046 |
| SLC39A6 | -0.562325349 | 0.00069187 |
| LKAAEAR1 | -0.517837922 | 0.000725656 |
| SPATS2 | -0.659269071 | 0.000728415 |
| TMSB15A | -0.59369687 | 0.000749501 |
| ZNHIT3 | -0.524745947 | 0.000770626 |
| EMC6 | -0.554287289 | 0.000772712 |
| CHD4 | -0.550147744 | 0.000774939 |
| RECQL | -0.577268754 | 0.000797227 |
| ATP1B1 | -0.6219321 | 0.000799584 |
| RABAC1 | -0.515201847 | 0.000806425 |
| FCGRT | 0.519219844 | 0.00080724 |
| MEST | -0.545106557 | 0.000811314 |
| CISD1 | -0.559007036 | 0.000837201 |
| COPZ1 | -0.614440463 | 0.000846696 |
| TEAD1 | -0.51506891 | 0.000853399 |
| MRPL14 | -0.56957216 | 0.000861452 |
| KLHL23 | -0.60310675 | 0.000862687 |
| CCDC91 | -0.503763423 | 0.000863128 |
| SPTBN1 | -0.501486028 | 0.000865039 |
| CMC2 | -0.589287436 | 0.000959384 |
| TULP4 | -0.52091918 | 0.000961793 |
| TPT1 | 0.680585157 | 0.000984055 |
| MSI2 | -0.60548424 | 0.001014837 |
| CENPQ | -0.541267143 | 0.001038699 |
| RHOB | 0.71266533 | 0.001069902 |
| NR2F1 | -0.78967922 | 0.001094503 |
| SNX6 | -0.535343865 | 0.001097414 |
| LINC00461 | -0.53873144 | 0.001131396 |
| SMAP2 | 0.500100829 | 0.001149644 |
| ZADH2 | -0.528717513 | 0.001174701 |
| ZFP36L1 | 1.175940657 | 0.001223673 |
| NFIX | -0.609676151 | 0.001249577 |
| DPP6 | -0.547322354 | 0.001304915 |
| AES | -0.516991294 | 0.001318712 |
| KIF2A | -0.537939142 | 0.001360733 |
| EMC9 | -0.54928349 | 0.001464765 |
| GTSE1 | -0.783544154 | 0.001476623 |
| CDKN1B | -0.609779287 | 0.001504932 |
| SUGP2 | -0.598870653 | 0.001516286 |
| KIF3A | -0.554377924 | 0.001526471 |
| PSMG4 | -0.579068205 | 0.001537755 |
| HNRNPUL1 | -0.548076104 | 0.00154192 |
| MKI67 | -0.786411432 | 0.001580624 |
| PLCB1 | -0.532973919 | 0.001611006 |
| PRPF4B | -0.533754886 | 0.001634983 |
| ZCCHC24 | -0.520944617 | 0.001662691 |
| TCEAL7 | -0.573911073 | 0.001745695 |
| TAOK1 | -0.540804299 | 0.001792698 |
| MAD2L1 | -0.509308782 | 0.001819449 |
| MYT1 | -0.557121594 | 0.001832571 |
| CENPF | -0.511701464 | 0.001854304 |
| RAB13 | 0.627164955 | 0.001887243 |
| CXADR | -0.578520791 | 0.001889156 |
| RGCC | -0.6041065 | 0.001908752 |
| CREB5 | -0.586244603 | 0.001921505 |
| GRN | 0.537522093 | 0.00194233 |
| EPS8 | -0.577882296 | 0.001957935 |
| CEP78 | -0.583076266 | 0.001966171 |
| PNRC1 | 0.501234499 | 0.00201396 |
| GRIA4 | -0.538254813 | 0.002051874 |
| ACADVL | -0.535948112 | 0.002076382 |
| CTNND2 | -0.570956588 | 0.002101801 |
| SYT11 | -0.51936161 | 0.002103538 |
| TROAP | -0.5912009 | 0.002145443 |
| SV2A | -0.556752769 | 0.002209722 |
| ITGB8 | -0.520916255 | 0.002233103 |
| MMP2 | -0.550830546 | 0.002298689 |
| MORF4L2 | -0.597289767 | 0.002318322 |
| TMEM132B | -0.559306397 | 0.002421277 |
| LMO1 | -0.559595588 | 0.002431904 |
| MLEC | -0.503661388 | 0.002501234 |
| C12orf45 | -0.528085103 | 0.002544492 |
| UQCC3 | -0.502999419 | 0.002584906 |
| GNAS | -1.252278424 | 0.002608055 |
| SOX10 | -0.578321125 | 0.002766937 |
| CDCA3 | -0.562048237 | 0.002782418 |
| ALCAM | -0.517371936 | 0.002862355 |
| ZMAT3 | -0.557001004 | 0.00296669 |
| MAGOHB | -0.503794076 | 0.003030662 |
| PIAS2 | -0.554677827 | 0.003120526 |
| ARL6IP1 | -0.843249656 | 0.003152429 |
| ARF3 | -0.571755893 | 0.003343212 |
| GNL1 | -0.536162138 | 0.003425625 |
| EMP2 | -0.585396876 | 0.003496186 |
| BBX | -0.559291096 | 0.003542542 |
| SOX4 | -0.866149536 | 0.003559216 |
| CSGALNACT1 | -0.522664426 | 0.003815518 |
| NUSAP1 | -0.772921511 | 0.003927621 |
| NT5C3A | -0.514985713 | 0.003965505 |
| RTL8C | -0.556113385 | 0.004058837 |
| BTG3 | -0.509994128 | 0.004062681 |
| RUFY3 | -0.516133045 | 0.004075006 |
| RASSF4 | 0.638823389 | 0.004083167 |
| GYPC | -0.553097533 | 0.004229805 |
| TSC22D3 | 0.805144451 | 0.004260815 |
| LPL | -0.609489031 | 0.004369203 |
| TPX2 | -0.655329708 | 0.004507205 |
| TRIB2 | -0.577174651 | 0.00450819 |
| PCMTD2 | -0.563453793 | 0.004673925 |
| CENPW | -0.521981184 | 0.004833918 |
| TMPO | -0.51271684 | 0.004982995 |
| RACGAP1 | -0.556566982 | 0.005347692 |
| S100A6 | 1.519670054 | 0.005803527 |
| NEAT1 | 1.976182617 | 0.00590614 |
| CRMP1 | -0.508327461 | 0.005937623 |
| NAXE | -0.572129653 | 0.006086797 |
| H2AFX | -0.626677843 | 0.006095846 |
| KPNA2 | -0.910817184 | 0.006134388 |
| KANK1 | -0.555487716 | 0.006612883 |
| CTSZ | 0.815928219 | 0.00690761 |
| TCEAL8 | -0.635996407 | 0.007174151 |
| ATF7IP | -0.514502786 | 0.007243143 |
| APOE | 1.687560048 | 0.007271136 |
| CAMK2N1 | -0.768442598 | 0.007728765 |
| LHFPL2 | 0.565474363 | 0.008039582 |
| AGAP1 | -0.53601572 | 0.008532037 |
| ATP2B1 | -0.568379547 | 0.008743212 |
| DECR1 | -0.511700228 | 0.008921265 |
| ZNF292 | -0.540251245 | 0.009191462 |
| KIF11 | -0.537743461 | 0.009337674 |
| SDF2L1 | -0.614811014 | 0.009422239 |
| PCM1 | -0.514671382 | 0.010114039 |
| VCAN | -0.604591164 | 0.010213942 |
| PDGFRA | -0.573171783 | 0.011565313 |
| FKBP5 | 0.601364808 | 0.01188958 |
| CTXN1 | -0.506331637 | 0.012354269 |
| CTSB | 0.682247407 | 0.012554635 |
| RAD21 | -0.582661944 | 0.013045426 |
| ZNF652 | -0.509581038 | 0.013638706 |
| CD82 | -0.92260771 | 0.014029067 |
| RTKN2 | -0.519428601 | 0.014523853 |
| BZW2 | -0.542340074 | 0.015431124 |
| SIRT2 | -0.587513542 | 0.015462257 |
| MPHOSPH8 | -0.535938661 | 0.015805161 |
| HIPK2 | -0.521426451 | 0.015896119 |
| GGCT | -0.541551438 | 0.016818087 |
| AKAP12 | -0.55940968 | 0.016828379 |
| EEF1E1 | -0.512151441 | 0.017339757 |
| CENPE | -0.67507715 | 0.018219041 |
| MAFB | 0.998331691 | 0.020671369 |
| LRRK2 | -0.523835753 | 0.021235844 |
| TRAF4 | -0.535986791 | 0.024144989 |
| ANXA6 | -0.524442475 | 0.024915762 |
| SGO1 | -0.539228295 | 0.025588299 |
| SLC44A1 | -0.539899168 | 0.026152035 |
| DDIT4 | -0.600355239 | 0.029225524 |
| ID4 | -0.640706269 | 0.030314927 |
| RNASET2 | 1.27007507 | 0.039689996 |
| AKR7A2 | -0.568223348 | 0.039995921 |
| DLGAP5 | -0.596911975 | 0.04090471 |
| LYRM4 | -0.53851034 | 0.041765737 |
| BICD1 | -0.508038142 | 0.047775942 |
